# Multifunctional Engineering-Enabled Electron Transport in SnO_2_ for Sn-Based Perovskite Solar Cells in the n‑i‑p Configuration

**DOI:** 10.1021/acsami.5c12227

**Published:** 2025-08-21

**Authors:** Parameswaran Rajamanickam, Ingita Tiwari, Leena Nebhani, Eric Wei-Guang Diau

**Affiliations:** † Department of Materials Science and Engineering, 34914 National Yang Ming Chiao Tung University, 1001 Ta-Hsueh Rd, Hsinchu 300093, Taiwan; ‡ Department of Materials Science and Engineering, 28817 Indian Institute of Technology Delhi, Hauz Khas, New Delhi 110016, India; § Department of Applied Chemistry and Institute of Molecular Science, 34914 National Yang Ming Chiao Tung University, 1001 Ta-Hsueh Rd, Hsinchu 300093, Taiwan; ∥ Center for Emergent Functional Matter Science, National Yang Ming Chiao Tung University, 1001 Ta-Hsueh Rd, Hsinchu 300093, Taiwan

**Keywords:** multifunctional engineering, tin perovskite solar cells, two-step method, polybenzoxazine, interfacial redox reaction, electron transport, back-surface field

## Abstract

The two-step sequential deposition technique reported in the inverted p-i-n configuration to fabricate Sn perovskite solar cells fails in the TiO_2_-based n-i-p configuration since the latter aggravates Sn^2+^ oxidation from the SnI_2_ nucleation layer upon pore infiltration. However, ambipolar SnO_2_ only promotes hole transport in Sn perovskite. Here, we report Cl-doped SnO_2_ (Cl:SnO_2_) with surface functionalities using multifunctional polybenzoxazine (p-Benz) to circumvent the SnO_2_/SnI_2_ interfacial redox reaction that would otherwise amplify hole extraction. The p-Benz functionalization altered the photoemissive properties of the transparent electrode and introduced a small charge transport resistance against undesirable carrier leakage toward the ETL side by simultaneously enabling contact establishment in the dark. When illuminated, the hole-rich Sn perovskite in contact with the PTAA hole-transport layer allows rapid hole injection, which induces an internal electric field, leading to a functioning planar n-i-p device. By integrating the two-step method in the n-i-p configuration and facilitating selective electron transport in SnO_2_, the versatility in device engineering is uncovered.

## Graphical abstract

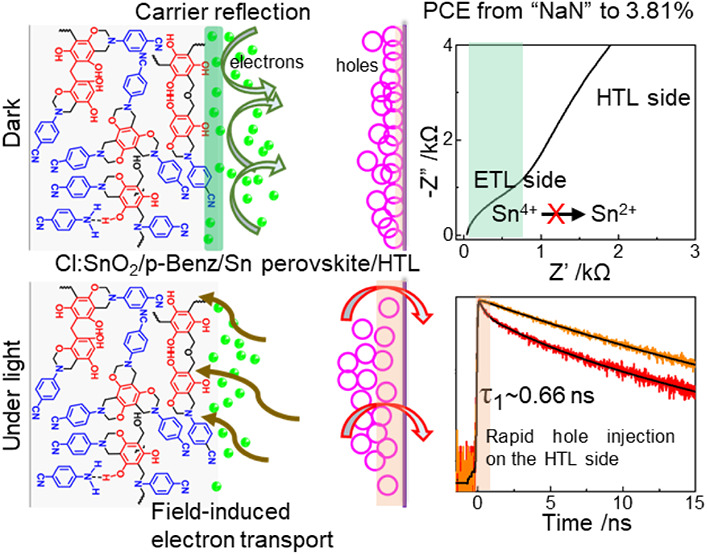

## Introduction

Lead-free tin-based perovskite solar cells (TPSCs) have witnessed burgeoning development over the years, reaching a power conversion efficiency (PCE) of 17.13%, thanks to the strategic implementation of the inverted p-i-n device configuration and 2D/3D heterostructure engineering.^1^ This rapid progress is primarily attributed to the widely used one-step antisolvent-dripping method.^2,3^ We introduced a two-step sequential deposition technique based on mixed solvents to slow down the perovskite crystallization process, leading to less-defective films.^4^ The technique is well established in the inverted device configuration, resulting in the development of hole-transport-material-free TPSCs.^5–8^ However, on the downside, successive spin-coating of SnI_2_ nucleation layer and organic iodide could aggravate Sn^2+^ oxidation in the nucleation layer, obstructing device fabrication in the regular n-i-p configuration that traditionally employs mesoporous TiO_2_ scaffolds to tune the optoelectronic properties of the tin-based perovskites.^9–12^ Hence, the two-step method has been unexplored thus far in the n-i-p device configuration.

Confronting this challenge encourages alternatives, such as tin­(IV) oxide (SnO_2_), characterized by superior stability in the UV region, better conductivity, and facile processability.^13^ While defective SnO_2_/perovskite interface were discussed in lead-based perovskite solar cells,^14–16^ strategies to overcome these issues exist.^17–20^ Nevertheless, the inevitable SnO_2_/SnI_2_ interfacial redox reaction inhibits electron transport in TPSCs,^21^ and hence its usage toward selective electron extraction has been rarely reported.^22^ Further studies attribute the presence of mixed-valent Sn species to the bipolar carrier transport of SnO_*x*_ ^23–25^ that requires intervention to facilitate desired carrier selection.

In this context, we report a double-reinforced SnO_2_/Sn perovskite interface in the n-i-p device configuration, where Sn perovskite is formed by a two-step method. The interfacial reinforcement was enabled by doping SnO_2_ with chlorine (Cl:SnO_2_),^26^ followed by surface functionalization using a newly synthesized multifunctional polybenzoxazine (p-Benz) via a facile immersion method. Introducing Cl at the buried interface was inspired from a recent finding,^27^ while the lack of a molecular electron transport layer (ETL)/perovskite interfacial modification strategy in the n-i-p configuration of TPSCs motivated the application of p-Benz functionalization.^28^

## Results and Discussion

Multifunctional p-Benz particles were synthesized with abundant N-functionalities and hydroxyls (Figure S1) to passivate perovskite buried interface defects and SnO_2_ (and Cl:SnO_2_) surface defects by strongly adhering to the metal oxide surface via hydrogen bonding.^29^ Its spherical morphology could suppress multilayer physisorption, while its highly cross-linked network was expected to tailor the photoemissive properties of the underlying transparent electrode, leading to controlled interfacial carrier transfer and transport. The cross-linked conformal networks of multifunctional p-Benz can not only passivate the metal oxide surface but also enhance the chemical, thermal, and interfacial stability. Its molecular flexibility, when dispersed in ethanol, allows the network’s favorable interaction with the polar protic solvent, inducing dynamic reorientation of functional groups while adhering to the metal oxide layer. This, in turn, regulates the interfacial interactions in situ.^30^ The reorientation driven by surface energy minimization enables directional alignment of polar groups (−OH, −NH_2_, −C≡N) toward the electrode, resulting in multisite functionalization. In contrast, traditional SAMs, with rigid molecular backbones and well-defined anchoring moieties/groups, lack such adaptive behavior and are prone to limited reconfiguration after initial adsorption, leading only to single-point functionalization.^31^ Crucial to device performance lies the quality of the substrate on which the photoactive layer grows. Hence, the bulk grainy, pinhole-rich F-doped tin oxide (FTO) glass surface (Figure S2) was first covered with a compact SnO_2_ layer by spin-coating colloidal nanoparticle dispersion. Adjusting the SnO_2_ suspension concentration, followed by bilayer deposition led to less-defective, compact, and uniform SnO_2_ film (Figure S3a,b).^32^ To obtain Cl:SnO_2_, NH_4_Cl was added to the diluted SnO_2_ colloid in the second step and spin-coated (refer to the Experimental Section). Subsequent functionalization of the electrode with p-Benz enriched the compact layer without forming a distinct layer (Figure S3c–f), which is critical for tailoring and exploiting the transparent electrode’s photoemissive properties. Overall, the compact nanoparticle layer inhibited diffuse reflectance on the substrate and improved the optical transparency of FTO/Cl:SnO_2_ (Figure S4) despite higher thickness (Figure S3b,d,f) arising from the multilayer coating. We observed that the perovskite films formed on FTO (Figure S5a) and FTO/p-Benz (Figure S5b) have poor morphology in comparison to the films formed atop the other electrodes (Figure S5c–f) due to the uneven, defective surface of the former.

The presence of Cl and N in Cl:SnO_2_ and Cl:SnO_2_/p-Benz was verified using XPS (Figures S6 and S7). While bare FTO could have adventitious N, the observed spectral broadening and peak downshift of the species in both FTO/p-Benz and SnO_2_/p-Benz (Figure S7a) result from amine- and nitrile-rich p-Benz functionalization.^33^ NH_4_Cl additive yields amino/imino- and protonated-amino-group signals on FTO/Cl:SnO_2_,^34^ whereas the latter gets replaced by cyano/trisubstituted nitrogen from the oxazine ring with excess amine functionalities from p-Benz in Cl:SnO_2_/p-Benz (Figure S7b). We observed substantial changes in the Sn species upon nanoparticle deposition and subsequent functionalization. The general trend in the Sn 3d_5/2_ core-level spectra acquired from the prepared electrodes is shown in Figure S8. Accordingly, FTO exhibits an asymmetrically broad peak, while a narrow peak with a slight upshift in peak maxima (∼486.5 eV) was observed for SnO_2_. The Cl:SnO_2_ film showed a significantly upshifted peak (∼486.8 eV) with almost no visible change in the peak width relative to SnO_2_ (Figure S8a). The Sn 3d spectra from FTO and FTO/p-Benz remained almost unaffected (Figure S8b), while the peak maxima moved toward higher binding energy for both SnO_2_/p-Benz (Figure S8c) and Cl:SnO_2_/p-Benz (Figure S8d) in comparison to their unfunctionalized counterparts. The asymmetric nature of the peak in bare FTO and FTO/p-Benz results from high degree of chemical compositional disorder, implying the presence of both SnO (Sn^2+^, ∼486.4 eV) and SnO_2_ (Sn^4+^, ∼487.3 eV) components, which agrees with our peak deconvolution (Figure S9).^35^ SnO_2_ colloidal nanoparticles, when spin-coated, behave as an additional electron source and transform the surficial SnO_2_ component on bare FTO into SnO, which manifested as a peak downshift and asymmetric-to-symmetric peak shape-shift. Hence, for the identical peak widths in the deconvolution parameters, we extrapolated only one component in the nanoparticle-covered electrodes, irrespective of the surface functionalities (Figure 1). For SnO_2_, the fitted peak corresponds to Sn^2+^ (∼486.5 eV, Figure 1a), while the fitted peaks in Cl:SnO_2_ (∼486.8 eV), SnO_2_/p-Benz (∼486.7 eV), and Cl:SnO_2_/p-Benz (∼487.0 eV) match with Sn^4+^ (Figure 1b–d).^36^ The overall peak-shifts in Sn^2+^ and Sn^4+^ components on surface-modified transparent electrodes with intact peak symmetricity are consequences of surface amorphization of the compact nanoparticle layer and modulation in the Madelung potential, which can alter the photoemission behavior.^37^ This peak shift on Cl:SnO_2_ and Cl:SnO_2_/p-Benz also implies charge transfer around Sn, resulting from its interaction with highly electronegative Cl and multifunctional p-Benz. Halogen ions like Cl, as n-type dopants in SnO_*x*_, replace the lattice oxides, modulate the electron density around Sn species, and create surface free electrons that ensure the dominance of SnO_2_, thereby leading to the observed changes.^38,39^ Also, the changes in nanoparticle-covered electrodes that are originally absent or negligible in bare FTO upon functionalization are ascribed to the abundance of dangling bonds on the nanoparticle surface. We further verified the SnO_2_ transformation with O 1s XPS spectra (Figure 2). The asymmetrically broad O 1s signal observed in FTO, Cl:SnO_2_, and Cl:SnO_2_/p-Benz shows a cumulative spectral upshift in peak maxima consistent with the changes in Sn species (Figure 2a). We deconvoluted the spectra from FTO into three components, each positioned at ∼530.1 eV, ∼531.3 eV, and 532.4 eV, respectively (Figure 2b), assigned to O–Sn^2+^, O–Sn^4+^, and loosely bound surface hydroxyls (M–OH).^40^ In contrast, deconvoluting the spectra from Cl:SnO_2_ and Cl:SnO_2_/p-Benz yielded only two components, assigned to O–Sn^4+^ and M–OH due to the observed cumulative peak shift (Figure 2c,d). This trend is in excellent agreement with our observation in Sn 3d XPS spectra, which accounts for increased SnO_2_ content rather than SnO_*x*_ (*x* < 2), conducive for electron transport.^40,41^

**Figure 1:**
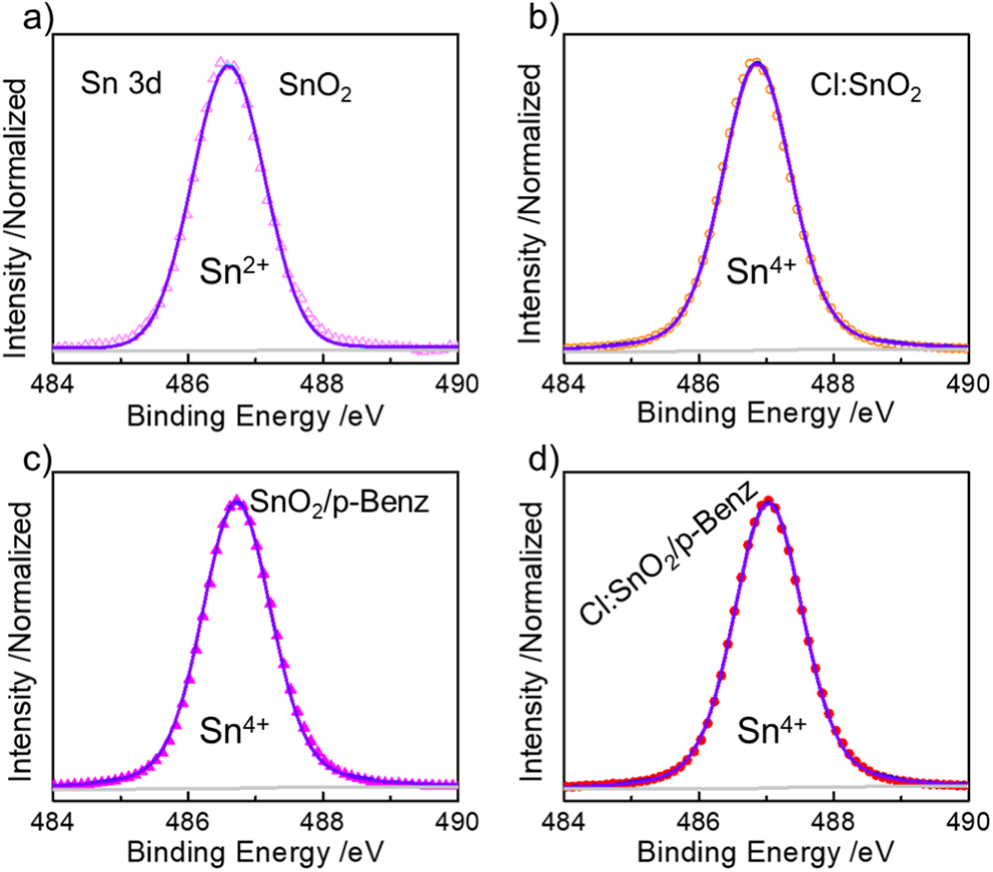
Deconvoluted high-resolution Sn 3d XPS spectra from a) FTO/SnO_2_, b) FTO/Cl:SnO_2_, c) FTO/SnO_2_/p-Benz, and d) FTO/Cl:SnO_2_/p-Benz showing a single peak coinciding with the cumulative fit peak as a result of FTO surface modification.

**Figure 2:**
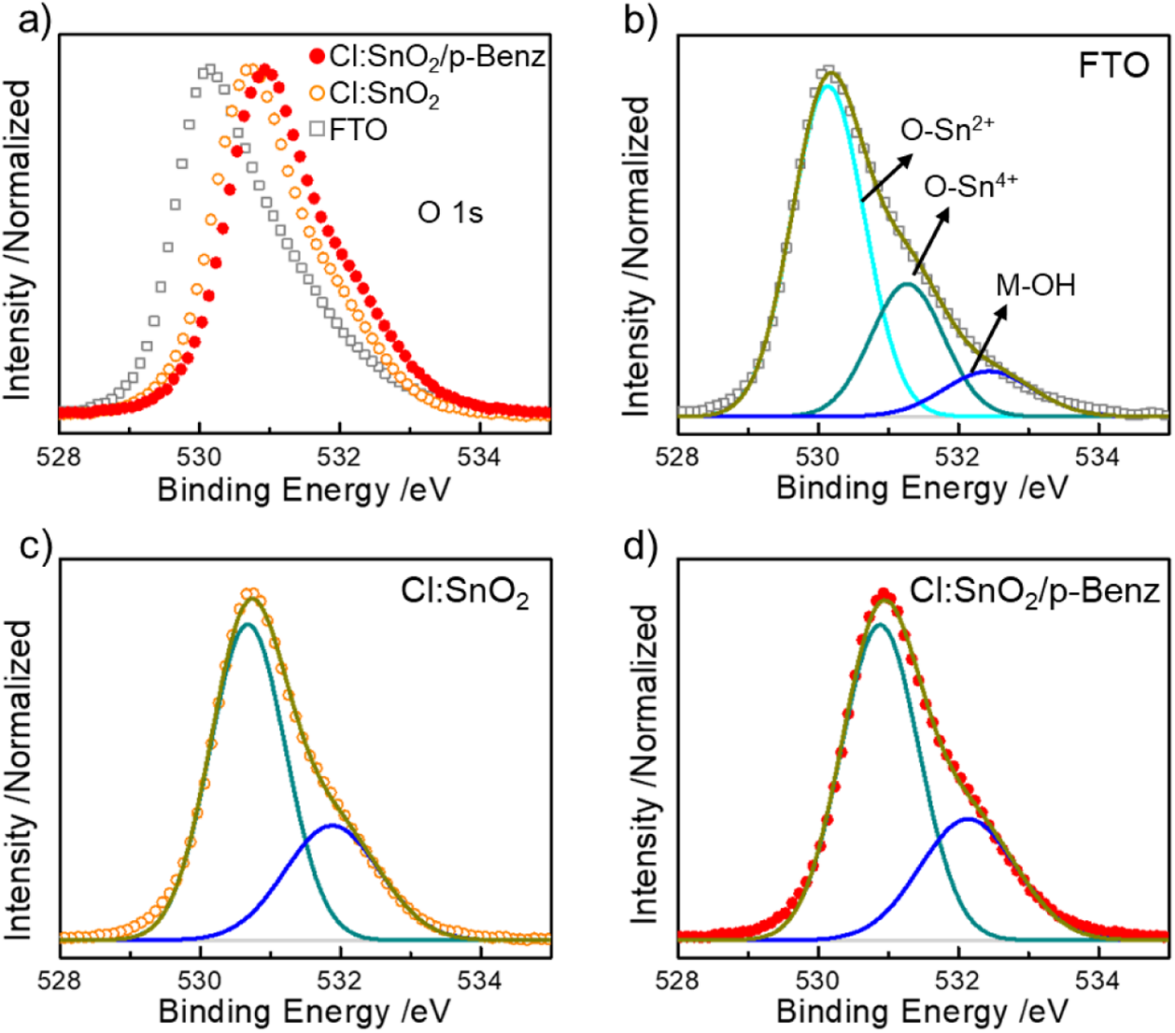
XPS of the O 1s from a) FTO, FTO/Cl:SnO_2_, and FTO/Cl:SnO_2_/p-Benz electrodes; Deconvoluted O 1s spectra from b) FTO, c) FTO/Cl:SnO_2_, and d) FTO/Cl:SnO_2_/p-Benz showing the contribution from the O–Sn^2+^, the O–Sn^4+^, and M–OH bonding. The overall peak shift between nanoparticle-covered electrodes and the bare substrate evidences the absence of surficial O–Sn^2+^ in the former.

We fabricated Sn-based perovskite solar cells using the prepared electrodes in the regular planar n-i-p configuration (FTO/ETL/p-Benz/Sn-perovskite/HTL/Au) with PTAA as the hole-transport layer (HTL) (Figure S10). The Sn perovskite layer was formed via two-step method as reported elsewhere,^4^ where the delayed injection of organic halide solution over SnI_2_ nucleation layer retards the perovskite crystallization rate as compared to the traditional one-step approach.^42^ The *J–V* characteristic curves from the best-performing devices under simulated illumination (AM 1.5G) are shown in Figure 3a,b with the cell parameters tabulated in Table 1. Accordingly, the device with a pure SnO_2_ layer showed no *J–V* characteristic, while the Cl:SnO_2_-based device displayed the observed *J–V* curve with a PCE of 1.95% (Figure 3a). The absence of an observable photovoltage in unfunctionalized SnO_2_-based device suggest abundant interfacial defects leading to ambipolar behavior of electrode-Sn species.^43^ However, both Cl and the residual N-species in Cl:SnO_2_ (vide XPS) passivated such defects to yield a *V*_OC_ of ∼0.260 V (Table 1).^26,44^ On the other hand, both p-Benz-functionalized electrodes facilitated and enhanced the device performance (Figure 3b). While the overall cell parameters have significantly improved, comparing both Cl:SnO_2_ and Cl:SnO_2_/p-Benz, *V*_OC_ and FF enhancement becomes evident in the latter (Table 1), suggesting superior interfacial characteristics.^45^ The high molecular dipole of the electrophilic nitrile groups in p-Benz inhibits a direct reaction between SnO_2_ and Sn perovskite, especially SnI_2_ after the first step of sequential deposition, which would otherwise form a possible homojunction that declines *V*_oc_, by favorably reacting with the interfacial Sn^4+^ via dipole–dipole and Lewis acid–base interactions. This mitigates the interfacial electron density accumulation and lowers the thermodynamic drive for Sn^4+^ to undergo chemical reduction. Furthermore, the presence of additional N– and −OH functionalities alter the ETL/perovskite interface to resist moisture infiltration via extensive intramolecular hydrogen bonding and passivate the interfacial oxygen vacancies by interacting through lone-pair donation with the ETL Sn species to further stabilize the Sn^4+^ oxidation state. This, overall, ensured *V*_oc_ and FF improvement in Cl:SnO_2_/p-Benz. Through UPS (Figures S11 and S12), UV–visible absorbance, and steady-state photoluminescence (PL) spectra (Figure S13) performed using the transparent electrodes, the colloidal nanoparticle dispersion, and the perovskite sample, the energy levels of the ETL and the perovskite layer were deduced (Figure S14). Irrespective of the surface functionalities, the SnO_2_ layers possess a deeper valence band maximum (VBM) making them suitable for blocking holes. The major difference is seen in Cl:SnO_2_ that has a relatively deeper conduction band minimum (CBM) alongside a much deeper VBM (Figure S14). Interestingly, the energy difference between the CBM and the Fermi level (*E*_f_) is much smaller in Cl:SnO_2_ and Cl:SnO_2_/p-Benz (0.06 eV), implying that the electrodes become more degenerate. This suggests heavy doping and strong surficial interaction between the Sn-, Cl-, and N-containing functionalities. Additionally, the electron-rich benzene rings and N-functional groups in p-Benz induce charge delocalization throughout the electrode surface, thereby modulating the energy levels. Nevertheless, we infer from the device characteristics in Figure 3a,b that the energy level alignment alone did not determine carrier extraction. The device *J*_SC_ from the *J–V* characteristics was authenticated by the integrated *J*_SC_ (∼16.991 mA cm^–2^) from the IPCE spectra (Figure 3c), and the device results are reproducible (Figure 3d). The *J–V* curves recorded in both reverse (*V*_OC_ to 0 V) and forward (0 V to *V*_OC_) directions show substantial hysteresis in SnO_2_/p-Benz, which becomes lower for Cl:SnO_2_ and negligible for Cl:SnO_2_/p-Benz (Figure S15). The presence of Cl at the ETL/perovskite interface can reduce the interfacial defect density and stabilize carrier flow,^26^ while the N-moiety from both NH_4_Cl and p-Benz synergistically manages both charge carriers and ion movement by trap passivation,^44^ suppressing hysteresis in Cl:SnO_2_/p-Benz (Figure S15b,c).

**Figure 3:**
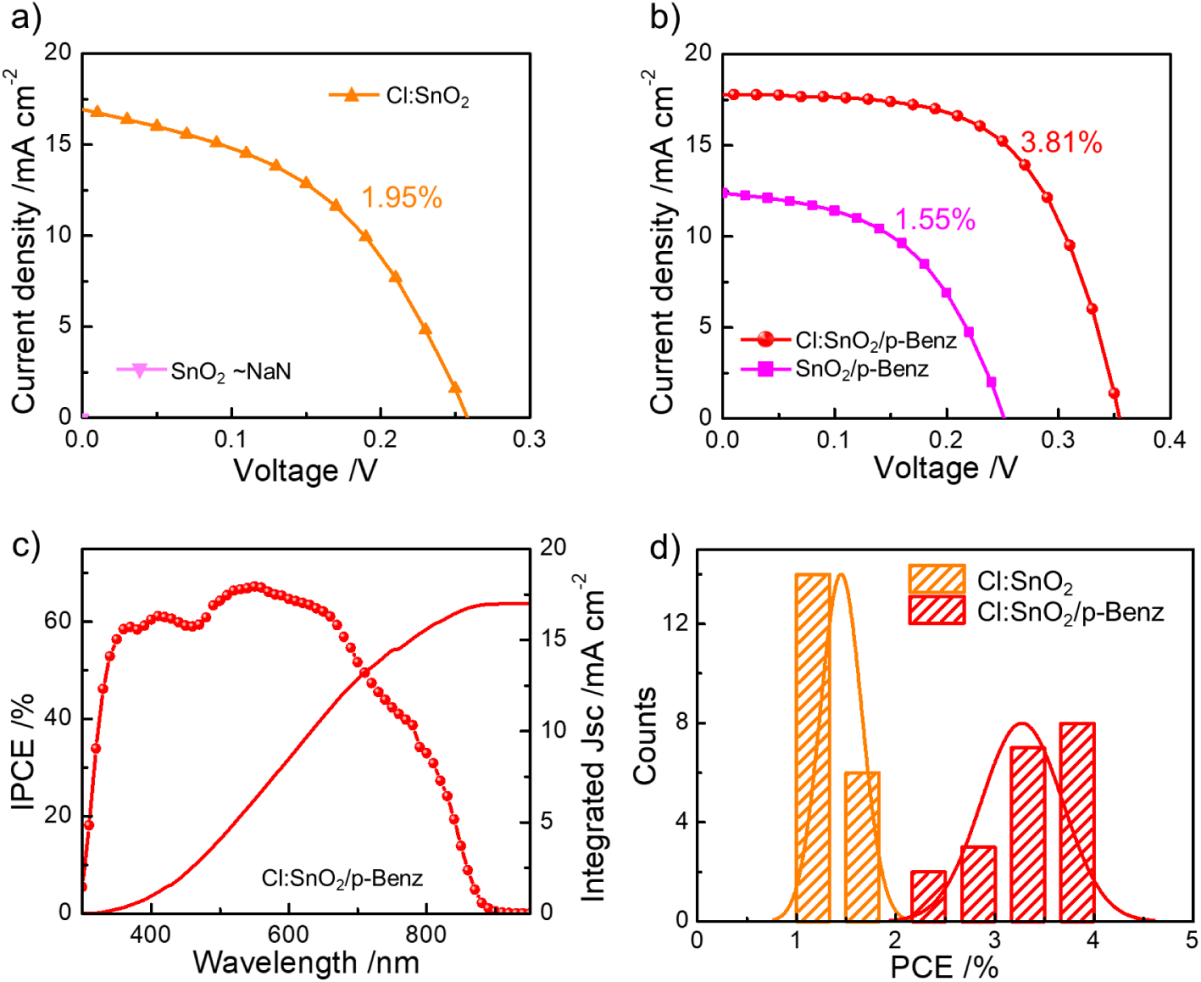
Representative *J–V* characteristic curves recorded from the devices using a) unfunctionalized, b) functionalized electrodes, c) IPCE spectra from FTO/Cl:SnO_2_/p-Benz-based device, and d) PCE histogram of 20 devices based on Cl:SnO_2_ and Cl:SnO_2_/p-Benz electrodes.

**Table 1:** Photovoltaic Cell Parameters Corresponding to the *J–V* Curves in Figure 3a,b

Device	*J*_SC_/mA cm^–2^	*V*_OC_/V	Fill Factor	PCE/%
SnO_2_/p-Benz	12.375	0.255	0.492	1.55
Cl:SnO_2_	16.951	0.260	0.444	1.95
Cl:SnO_2_/p-Benz	17.824	0.356	0.601	3.81

We performed dark *J–V* characteristics (Figure S16a) and estimated the lowest dark saturation current density from Cl:SnO_2_/p-Benz (∼0.11 μA cm^–2^ vs ∼1.05 μA cm^–2^ for Cl:SnO_2_). The relatively steeper carrier extraction slope observed in Cl:SnO_2_/p-Benz under forward bias suggests lower background carrier and trap density, leading to subdued carrier recombination away from the depletion zone.^46^ A simple equivalent circuit diagram of a solar cell based on the single diode model is shown in Figure S16b with parasitic resistances to account for the electrical losses. The series resistance (*R*_s_) impacts the ohmic losses surrounding the front and rear contacts (metal/semiconductor junction), and the shunt resistance (*R*_sh_) reflects carrier leakage and shunt pathways. We estimated a 10-fold higher *R*_sh_ (∼1173.71 Ω cm^2^ vs ∼119.05 Ω cm^2^) and a relatively lower *R*_s_ (∼2.92 Ω cm^2^ vs ∼3.61 Ω cm^2^) for Cl:SnO_2_/p-Benz device than the Cl:SnO_2_ device which ascertain managed carrier losses in the device, engendering *V*_OC_ and FF enhancement. We further probed the carrier transport dynamics under darkness using electrochemical impedance spectroscopy (EIS). The EIS Nyquist plots shown in Figure 4a display characteristics associated with electronic displacement and dipolar relaxation at the high- and intermediate-frequency regime (4 MHz to 1 Hz) and ionic contact phenomena and electrochemical processes at low frequency regime (<1 Hz).^47^ We chose to investigate the spectra recorded at short-circuit conditions (0 V) so as to unravel the role of p-Benz in directing and facilitating carrier flow. The spectra at zero bias also reflects the effect of *R*_sh_. From the semicircular arc-shaped spectra as opposed to a purely capacitive spectral signature, we inferred that the carrier transport layers are not perfectly blocking.^48^ We fitted the Nyquist plots using appropriate equivalent circuit models (Figure S17a,b and Table S1)^49^ to quantify and physically interpret the observed spectra. Accordingly, a simple parallel RC element fits the semiarc,^50^ and the spectral offset from the origin in the complex plane corresponds to the series resistance (*R*_s_) in the device. The arc diameter is influenced by both impedance against carrier transport (*R*_tr_) and recombination (*R*_rec_). We modeled the large semicircular spectra in Cl:SnO_2_ with a single RC component connected to *R*_s_ (Figure S17a) and assigned to the impedance against carrier recombination (Table S1). Whereas, the spectra from Cl:SnO_2_/p-Benz was modeled with an additional RC element (Figure S17b) to account for the small arc present in the high frequency domain (Figure 4b) associated with the impedance against carrier transport (Table S1).^49^ This is the characteristic Warburg component that signifies control over carrier transport due to p-Benz functionalization. The carrier delocalization at the ETL/Perovskite interface created by multiple functionalities in the cross-linked p-Benz network structure activates the interfacial barrier against rapid carrier diffusion, leading to the manifestation of this additional element. Note that this feature is subtle, followed by a very large semicircle encompassing the intermediate- to low-frequency regimes suggesting longer diffusion length according to Diffusion–Recombination model.^51^ Overall, the recombination resistance is substantially higher in Cl:SnO_2_/p-Benz than in Cl:SnO_2_ (Table S1) with a time constant over 5 times larger than that in the former, which is crucial for superior device performance. The presence of carrier transport resistance is typically undesirable in a conventional device, especially if the device is studied under no-bias condition. However, owing to the inherent nature of the device architecture (n-i-p), the influx of electrons toward FTO could chemically reduce the Sn species (Sn^4+^ to Sn^2+^) atop the transparent electrode eventually, thereby compromising its electron-conducting properties in the longer term. Therefore, the presence of this minor carrier transport resistance in Cl:SnO_2_/p-Benz is pivotal since it ensures restrained carrier leakage (Figure S16a) and suppressed interfacial SnO_2_/Sn perovskite redox reaction (Figure 4b). This is further evidenced by the corresponding capacitance versus frequency plot (Figure S17c). Partitioning Figure S17c into 3 regions with respect to frequency, we observe a capacitance plateau region in the high- to intermediate-frequency regime (region II) that shows greater capacitance in Cl:SnO_2_/p-Benz device than in Cl:SnO_2_ by virtue of carrier trapping/blocking at the transport-layer/perovskite interface due to the presence of multifunctional p-Benz.^52^

**Figure 4:**
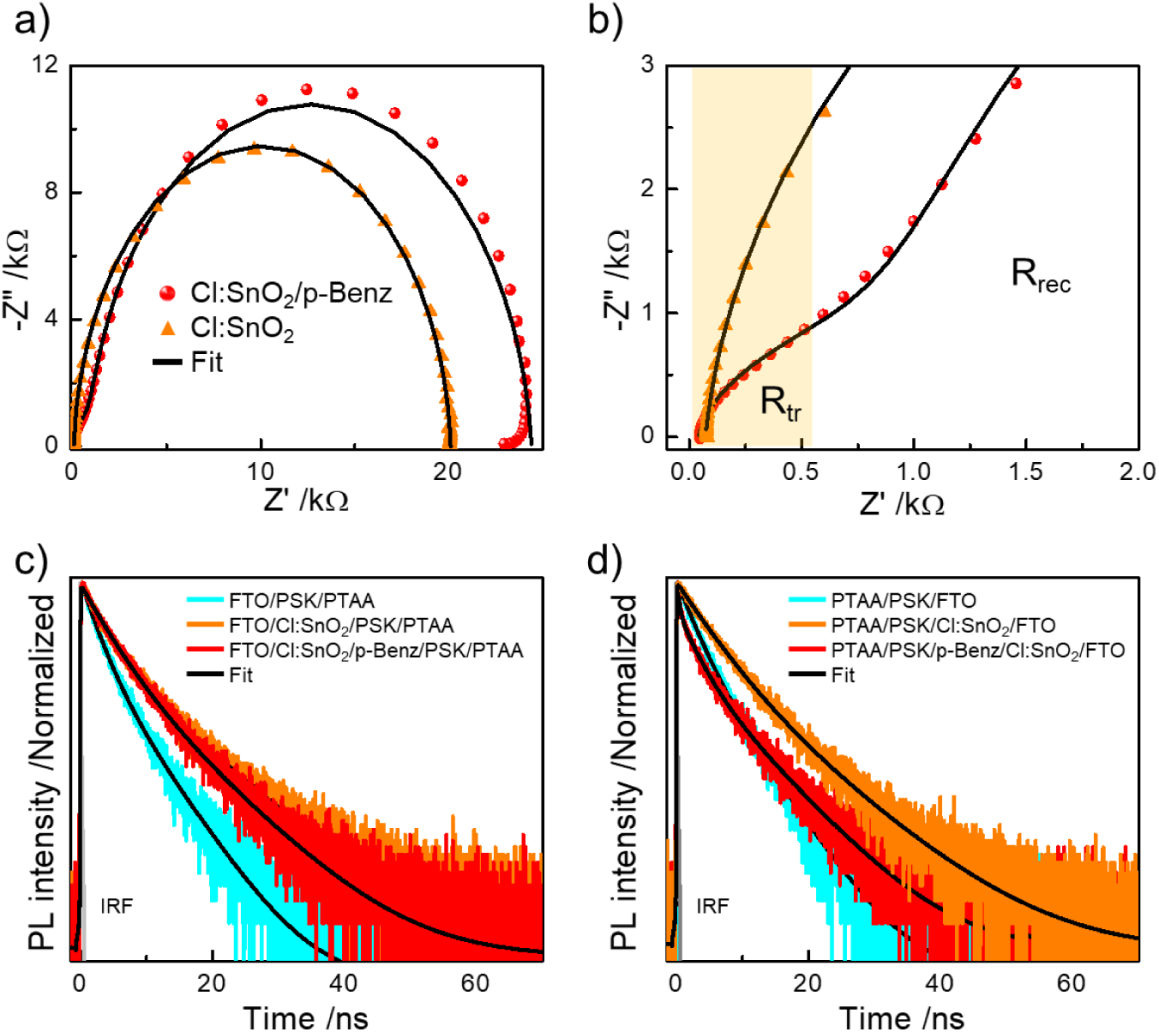
(a) EIS Nyquist plot (full range) and (b) its magnified view (near the high-frequency regime) recorded under darkness at short-circuit conditions; PL decay transients from the samples prepared on FTO, Cl:SnO_2_, and Cl:SnO_2_/p-Benz with PTAA were excited from (c) FTO side and (d) sample side, respectively.

We then examined the interfacial carrier dynamics upon photogeneration based on time-resolved PL spectra acquired using a time-correlated single-photon counting (TCSPC) technique. The decay transients were recorded at the maximum PL intensity of the samples by exciting them from both the transparent electrode side (Figure S18a) and the thin-film side (Figure S18b) to unravel the nature of carrier recombination.^53^ The transients were mostly fitted using biexponential decay functions (Tables S2 and S3) irrespective of the excitation side (Figure S18). The fast-decay lifetime component (τ_1_) results from trap-mediated recombination, and the slow-decay component (τ_2_) is assigned to radiative recombination.^54^ When compared to FTO/PSK, Cl:SnO_2_/PSK and Cl:SnO_2_/p-Benz/PSK exhibit substantial lifetime enhancement (Figure S18, Tables S2 and S3), most likely due to the superior perovskite surface morphology (Figure S5) of the latter. Note that both Cl:SnO_2_/PSK and Cl:SnO_2_/p-Benz/PSK possess almost similar average carrier lifetimes when excited from the thin-film side (Table S3 and Figure S18b) attesting to this aspect. However, when excited from the electrode side (Figure S18a), the longer lifetime relative to FTO/PSK is due to the buried-interfacial defect passivation effect of Cl- and N-rich p-Benz. Nevertheless, direct contact between the conductive electrode and perovskite is anticipated to quench the lifetime^6^ as seen in Cl:SnO_2_/p-Benz/PSK arising from uncontrolled carrier injection.

The persistence of the biexponential decay trend across all the samples with only subtle changes between Cl/SnO_2_ and Cl:SnO_2_/p-Benz could lead to possible ambiguity in our interpretation. Hence, we also performed TCSPC for the samples covered with HTL to assess the interfacial quality in the sample stack (Figure 4c,d and Tables S4, S5).^54^ The HTL brings in an additional carrier relaxation pathway for hole extraction, and with both ETL and HTL, a comprehensive interpretation can be established to complement the EIS result. Overall, even with HTL, prolonged carrier lifetime was mostly observed with biexponential decay under all the conditions except when the Cl:SnO_2_/p-Benz-based sample was excited from the HTL side (Figure 4d). We fitted PTAA/PSK/p-Benz/Cl:SnO_2_/FTO decay curve using a triexponential function (Table S5) and noticed a third rapid decay component with a very short lifetime of ∼0.66 ns in addition to the previously observed components with lifetimes of 4.34 ns (trap-mediated) and 9.13 ns (radiative recombination). The average carrier lifetime was estimated to be 5.40 ns, which is much closer to that observed in FTO-based samples. Since the device using Cl:SnO_2_/p-Benz displayed superior performance, the observed additional rapid relaxation is assigned to hole injection from Sn perovskite to HTL. In short, Cl:SnO_2_/p-Benz electrode led to buried interface passivation by virtue of Cl- and N-rich environment, yielding prolonged carrier lifetime, while PTAA as HTL in direct contact with Sn perovskite enabled swift hole-carrier extraction, thereby reducing the average carrier lifetime. With Au as the metal electrode, this resulted in a functioning Sn perovskite device in the regular planar n-i-p configuration. Hence, a complementary behavior is observed between the carrier-extracting layers as discussed using both EIS and TCSPC.

Based on our observations, we propose a simple mechanism of charge transport, as illustrated in Figure 5. In the absence of illumination, upon the establishment of contact between different layers, charge carriers undergo redistribution and reach equilibrium. Excess carriers can leak through interfacial defects to the adjacent carrier-transport layers, inducing redox reactions at the ETL/Sn perovskite interface. This is circumvented by p-Benz in Cl:SnO_2_/p-Benz (Figure 5a) as discussed using dark *J–V* characteristics (Figure S16) and EIS (Figure 4a). Upon photoexcitation, the hole-rich Sn perovskite allows photogenerated holes to distribute exponentially along the depth of the device stack, starting from the HTL/perovskite interface. Hence, holes are preferably, and swiftly, extracted toward the HTL, as observed in Figure 4d via TCSPC. This forms an internal electric field at the HTL/perovskite interface (back-surface field) that drifts and induces selective electron transport toward the FTO via Cl:SnO_2_/p-Benz (Figure 5b).

**Figure 5:**
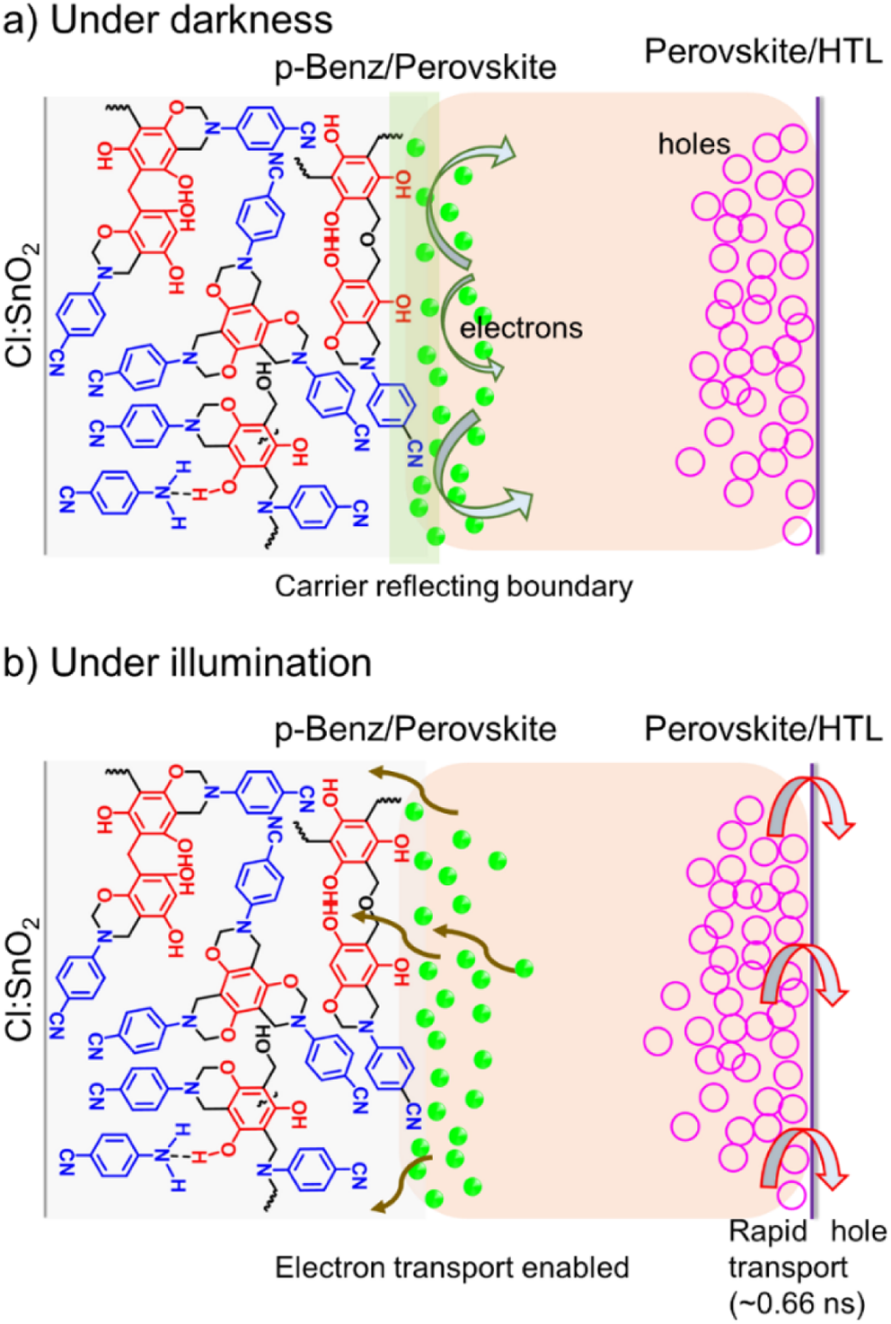
Graphical depiction of the charge transport mechanism in Cl:SnO_2_/p-Benz device under a) darkness and b) illumination. Under dark conditions, the solar cell behaves like a PN-junction diode with the charge carriers organizing close to the appropriate carrier-transport layers and reaching equilibrium while Cl:SnO_2_/p-Benz forms a carrier-reflecting boundary (vide EIS). When illuminated, hole-rich Sn perovskite in contact with the HTL hastens hole transport (τ_1_ ∼ 0.66 ns, vide TCSPC), inducing a back-surface field effect that ensures electron drift toward the ETL. Here, the p-Benz region (graphically exaggerated) enables electrons to tunnel through the barrier.

We tested the long-term stability of the best devices by intermittently measuring their *J–V* characteristics. The corresponding normalized device PCE evolution over time is shown in Figure S19a. Accordingly, Cl:SnO_2_ device lost over 60% of its initial efficiency within 3 days of fabrication, while the Cl:SnO_2_/p-Benz device was able to withstand for a relatively longer duration, retaining about 80% of the initial PCE for about 24 days. The storage stability primarily relies on the interfacial quality of the device stack,^6^ and the superior durability of Cl:SnO_2_/p-Benz device is attributed to the suppressed ETL/perovskite interfacial redox reaction, as elucidated using dark *J–V* characteristics and EIS. We further compared the performance of the devices fabricated using the designed electrodes by applying the traditional antisolvent-meditated one-step approach with that of the two-step approach. The *J–V* characteristic curves in Figure S19b display the superiority of sequential deposition technique over the one-step method (3.81% for two-step vs 1.66% for one-step), aligning with recent reports on the widely explored inverted p-i-n device configuration.^6,55,56^ Note that the performance trend remains identical with respect to the electrodes, viz., Cl:SnO_2_/p-Benz (1.66%) > Cl:SnO_2_ (0.87%) irrespective of the technique adopted. This is because of the electrode/perovskite buried-interfacial characteristics and chemical functionalities that not only initiate but also regulate the bottom-up growth of Sn perovskite from the SnI_2_ nucleation layer in the two-step method that is originally lacking in the one-step method.

## Conclusion

In conclusion, we fabricated Sn perovskite solar cells via a two-step method for the first time in a regular planar n-i-p configuration. The fabrication was aided by modifying the surface characteristics of SnO_2_-based ETL. Surface doping of SnO_2_ with Cl and subsequent functionalization (p-Benz) circumvented the SnO_2_/Sn perovskite interfacial redox reaction that would chemically reduce the interfacial Sn species from Sn^4+^ to Sn^2+^, which promotes hole transport. Our strategy tailored the photoemissive properties of the ETL, leading to selective electron extraction, which has never been observed in SnO_2_ ETL when employed alongside lead-free TPSC. The vital role of p-Benz functionalization in mitigating the undesirable ETL/perovskite interfacial reaction was clarified via dark characterizations, while the photoexcited carrier kinetics revealed the almost equal role played by hole transport along the HTL side, which induced a back-surface field effect to enable a functioning n-i-p device. As a result, the device fabricated by using the new ETL (Cl:SnO_2_/p-Benz) gave rise to a PCE of 3.81%. While the performance is substantially low in comparison to the state-of-the-art, the method explored in this study reveals the versatility in Sn perovskite device engineering across architectures and the less-explored route toward optimizing SnO_2_ ETL. We further expect this interesting multifunctional engineering strategy to inspire future research toward improving device fabrication materials and methods, not only limited to lead-free perovskite solar cells.

## Experimental Section

### Materials

Tin iodide (SnI_2_, 99.999%, Aldrich), tin fluoride (SnF_2_, 99%, Aldrich), ethane-1,2-diammonium bromide (EDABr_2_, ≥98% Greatcell Solar), formamidinium iodide (FAI, >99.99%, Greatcell Solar), dimethyl sulfoxide (DMSO, Aldrich), chlorobenzene (CB, 99.8% anhydrous, Aldrich), isopropyl alcohol (IPA, 99.5% anhydrous, Aldrich), 1,1,1,3,3,3-hexafluoro-2-propanol (HFP, > 99%, TCI), tin­(IV) oxide (SnO_2_) aqueous colloidal nanoparticle dispersion (15% by weight, Alfa Aesar), ammonium chloride (NH_4_Cl, Honeywell), and Poly­[bis­(4-phenyl)­(2,4,6-trimethylphenyl)­amine] (PTAA, Luminescence Technology), phloroglucinol (>99.0%, TCI), 4-aminobenzonitrile (>98.0%, TCI), and formaldehyde solution (37–40 wt %, ThermoFisher Scientific Chemicals) were used as received. Anhydrous ethanol was dry-distilled prior to the dispersion of the synthesized polybenzoxazine (p-Benz) oligomer nanoparticles.

### Synthesis of 4-Aminobenzonitrile-Derived Polybenzoxazine Particles (p-Benz)

A detailed synthesis procedure for 4-aminobenzonitrile-derived polybenzoxazine particles (p-Benz) will be reported elsewhere. Briefly, phloroglucinol (1.6 mmol) and 4-aminobenzonitrile (4.8 mmol) were dissolved in a mixture of ethanol and DI water (320 mL, 1:3 v/v) under continuous stirring at 25 °C. After complete dissolution, formaldehyde solution (12.8 mmol) was added dropwise over 30 min, and the mixture was stirred for another 1 h at the same temperature. Subsequently, the reaction mixture was heated to 80 °C and maintained at that temperature for 24 h. The resulting particles were isolated by centrifugation (8000 rpm, 10 min), washed three times with DI water, and dried under vacuum at 50 °C.

### FTO Surface Modification

Commercially available prepatterned Fluorine-doped Tin Oxide (FTO) glass substrates (1.9 × 1.9 cm^2^) were washed via ultrasonication in a mixture of acetone, isopropyl alcohol, and DI water for 30 min, followed by oven-drying. The aqueous colloidal SnO_2_ nanoparticle dispersion was diluted with DI water in ratios of 1:5 (v/v) and 1:10 (v/v), respectively. Following a 30-min ultrasonic treatment, the 1:5 diluted nanoparticle dispersion and 1:10 diluted nanoparticle dispersion were filtered and spin-coated (3000 rpm, 30 s) successively atop the UV-ozone-treated (20 min) FTO glasses with intermittent annealing (160 °C, 20 min) in air. This successive deposition with controlled dilution ensures complete coverage of the compact SnO_2_ layer and yields FTO/SnO_2_ electrodes. To dope SnO_2_ with chlorine, ∼2.5 mg of NH_4_Cl was dissolved in the diluted SnO_2_ (1:10) dispersion via magnetic stirring at room temperature for 2–3 h.^1^ Subsequently, the solution was filtered and spin-coated atop FTO/SnO_2_ electrodes (3000 rpm, 30 s), followed by annealing (160 °C, 20 min) in air. We denote the resulting electrodes as FTO/Cl:SnO_2_. To functionalize the electrodes, ∼1 mg of the synthesized p-Benz nanoparticles was dispersed well in ∼20 mL of dry-distilled ethanol (enough to accommodate 6 electrodes at a time), and UV-ozone-treated (30 min) electrodes were immersed in the p-Benz bath for an overnight duration (∼8–12 h) at room temperature. Eventually, the electrodes were rinsed thoroughly and annealed on a hot plate at 120 °C for 15 min and moved to a nitrogen-filled glovebox for perovskite deposition. The functionalized electrodes are denominated as SnO_2_/p-Benz (functionalized FTO/SnO_2_) and Cl:SnO_2_/p-Benz (functionalized FTO/Cl:SnO_2_).

### Sn Perovskite Precursor and Film Formation

The perovskite ink was prepared in a nitrogen-filled glovebox. For the two-step sequential deposition method,^2^ the first step involves forming the SnI_2_ nucleation layer on the transparent substrates via spin-coating (40 μL precursor solution, 6000 rpm, 60 s without subsequent annealing). The SnI_2_ solution was prepared by dissolving SnI_2_ (∼372.5 mg), SnF_2_ (∼31.3 mg), and EDABr_2_ (∼11 mg) in DMSO (1.25 mL) at room temperature inside the glovebox. The second step involves spin-coating 100 μL of FAI solution (30 mg dissolved in a solvent mixture of IPA:HFP:CB in a ratio of 5:5:2) injected over the nucleation layer (5000 rpm, 12 s; spun after waiting for 40 s). Subsequently, the perovskite films were annealed at 90 °C for 15 min on a hot plate. PTAA (25 mg/mL in CB; stirred overnight), as the hole-transport layer, was formed atop the Sn perovskite layer via spin-coating (3000 rpm, 30 s) followed by annealing at 80 °C for 10 min. Finally, Au as the metal electrode (∼70 nm) was deposited via thermal evaporation under ultrahigh vacuum (pressure 5 × 10^–6^ Torr) to fabricate the device in the n-i-p configuration.

### Sample and Device Characterization

The FT-IR spectra of the synthesized p-Benz particles were acquired from NICOLET, iS50 (ThermoFisher Scientific). The electron microscopic images of the transparent electrodes and the perovskite films were captured using a Jeol JSM-7800F Prime Field Emission Gun Scanning Electron Microscope (SEM). X-ray photoelectron spectra (XPS) and ultraviolet photoelectron spectra (UPS) were acquired from a Thermo K-ALPHA Surface Analyzer. The XPS spectra were calibrated using C 1s spectra centered at 284.8 eV, while the calibration of the UPS spectra was based on the Fermi edge of standard Au to compensate for any existing mismatch in the spectra. For the UPS measurement, the samples were biased at −5 V to facilitate photoemission from the sample surface. A UV–visible spectrophotometer (V-780, Jasco) equipped with an integrated sphere (ISN-9011, Jasco) was employed to record the light transmittance and reflectance spectra, while the steady-state photoluminescence (PL) spectra were obtained using a laboratory-built PL system, as described elsewhere.^3^ For the PL measurement, the perovskite samples were optically excited at 450 nm, and the emission spectra were monitored between 600 and 1100 nm by blocking the scattering excitation signal using a long-pass filter (650 nm) at the entrance slit of the emission monochromator. The time-resolved PL spectra were recorded using a time-correlated single-photon counting (TCSPC) system (Fluotime 200, PicoQuant, fwhm ∼ 70 ps). The samples for the photophysical studies were prepared atop the transparent electrodes, followed by careful encapsulation with a glass substrate by applying optical adhesive (Norland 61, Thorlabs NOA61) and curing it using intense UV light illumination. The recorded transients were fitted using bi- and triexponential fitting functions with Fluofit software, and the average carrier lifetimes were estimated by applying the intensity average method (τ_avg_ = ∑i=13αiτi2∑i=13αiτi ).

All of the device measurements were performed under ambient conditions without device encapsulation. The *J*–*V* characteristic curves of the fabricated devices were recorded with the help of Keithley 2400 digital source meter under 1-sun illumination (AM 1.5G, 100 mW cm^–2^) using a solar simulator (XES-40S1, SAM-E1) calibrated with a standard reference silicon cell (Oriel, PN 91150 V, VLSI standard). The measurement was performed in both the reverse (open-circuit to short-circuit) and forward (short-circuit to open-circuit) directions with a voltage scan step of 0.02 V by applying a metal mask with an exposure area of 0.0225 cm^2^. Incident Photon-to-Current Conversion Efficiency (IPCE) of the devices was tested using QE-R Quantum Efficiency Measurement system (Enli Technology) after calibrating it with a standard Si detector (RC-S103011-E, Enli Technology). Electrochemical Impedance Spectroscopy (EIS) was performed in darkness by applying an AC amplitude of 10 mV to the voltage in the frequency range between 4 MHz and 0.1 Hz, with the devices being biased at 0 V (short-circuit condition). To obtain the corresponding capacitance vs frequency plot, we calculated the capacitance from the imaginary part of the impedance from the Nyquist plot using *C* = −(1/(2π*fj*) × (1/*Z*″), where *f* is the perturbation signal frequency.

## Supplementary Material



## Notes

Experiments; FT-IR, SEM, XPS, optical transmittance, UV–visible absorbance/PL spectra, UPS, device *J*–*V* characteristic curves (dark and illuminated), TCSPC, EIS, capacitance vs frequency plot, and fitting information (PDF)
