# Supplementary material for: Multifunctional Engineering-Enabled Electron Transport in SnO2 for Sn-Based Perovskite Solar Cells in the n‑i‑p Configuration
Source: ACS Appl Mater Interfaces. 2025 Aug 21;17(35):49584–93. doi: 10.1021/acsami.5c12227 (PMC12412096; doi:10.1021/acsami.5c12227)
Supplement: Supplementary file 1 [file am5c12227_si_001.pdf]

Supporting Information

Multifunctional-Engineering-Enabled Electron Transport in SnO_2 for Sn-based Perovskite Solar Cells in the n-i-p Configuration

Parameswaran Rajamanickam,[a] Ingita Tiwari,[b] Leena Nebhani,[b] Eric Wei-Guang Diau[c,d]*

[a]Department of Materials Science and Engineering, National Yang Ming Chiao Tung University, 1001 Ta-Hsueh Rd., Hsinchu 300093, Taiwan

[b]Department of Materials Science and Engineering, Indian Institute of Technology Delhi, Hauz Khas, New Delhi – 110016, India

[c]Department of Applied Chemistry and Institute of Molecular Science, National Yang Ming Chiao Tung University, 1001 Ta-Hsueh Rd., Hsinchu 300093, Taiwan

[d]Center for Emergent Functional Matter Science, National Yang Ming Chiao Tung University, 1001 Ta-Hsueh Rd., Hsinchu 300093, Taiwan

*Corresponding Author (E-mail: diau@nycu.edu.tw)

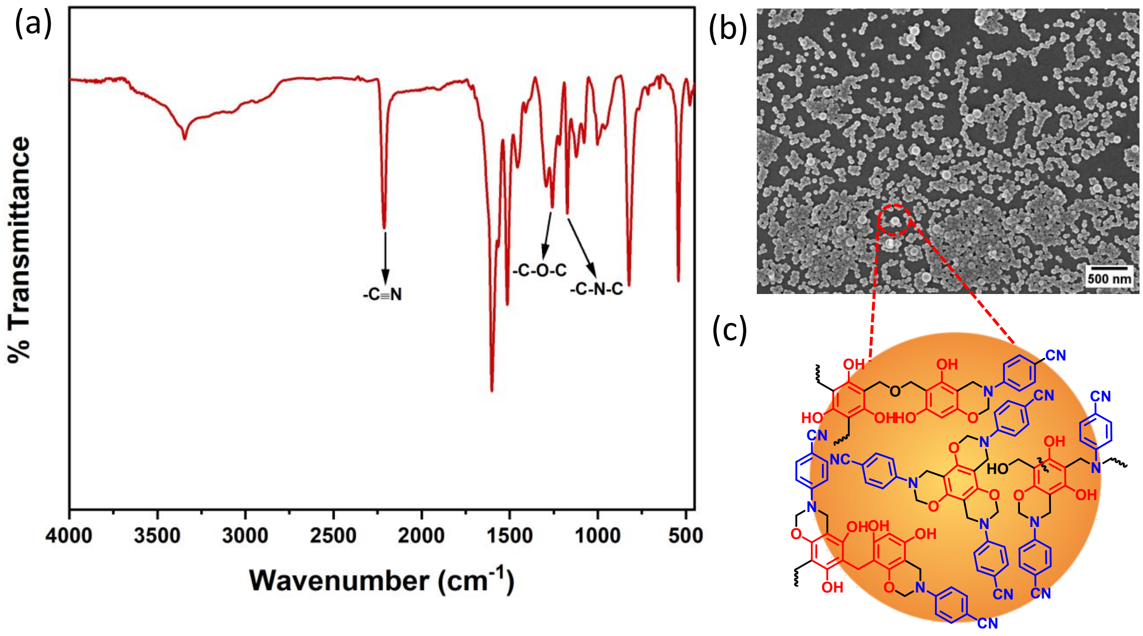

Figure S1. (a) FTIR spectra of p-Benz showing the functional groups present; (b) FESEM micrograph of the p-Benz particles (Avg. size = 104 ± 5.4 nm); and (c) Schematic representation of highly crosslinked p-Benz network.

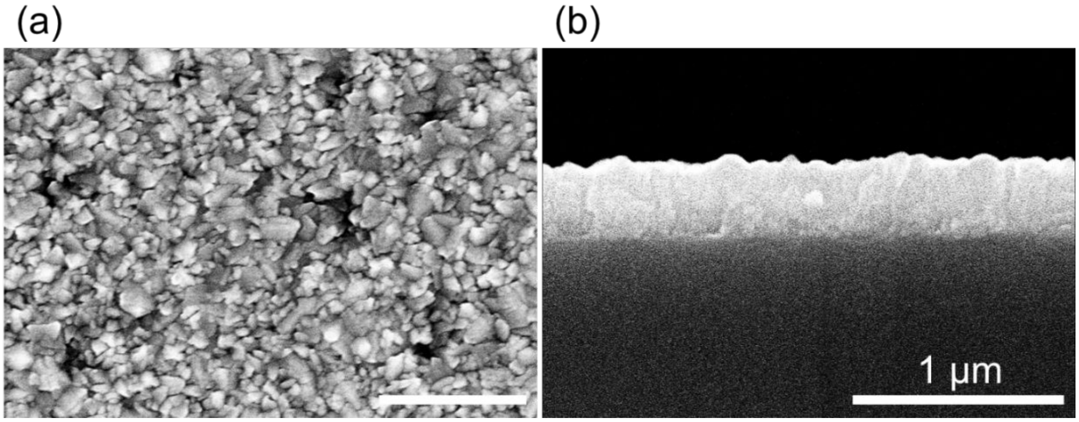

Figure S2. SEM (a) top-view and (b) cross-section of FTO glass substrate showing defect-rich surface and uneven cross-sectional morphology. The scale bars in both the figures correspond to 1 μm.

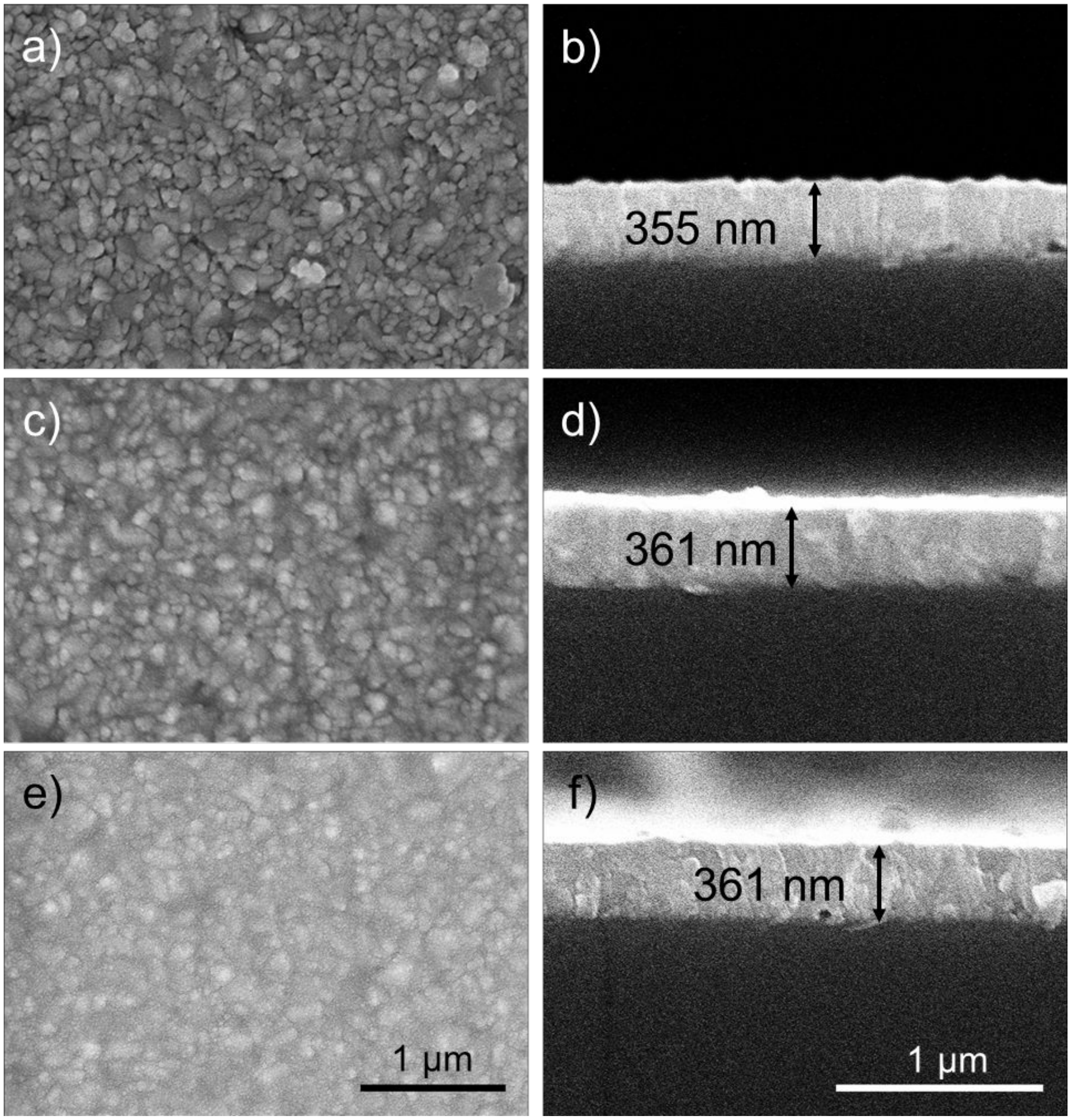

Figure S3. SEM top-view and cross-section, respectively, of (a,b), FTO/SnO_2 (c,d) FTO/Cl:SnO_2, and (e,f) FTO/Cl:SnO_2/p-Benz. The surface of the nanoparticle-coated layers is seen to be less defective and more compact with better cross-sectional uniformity.

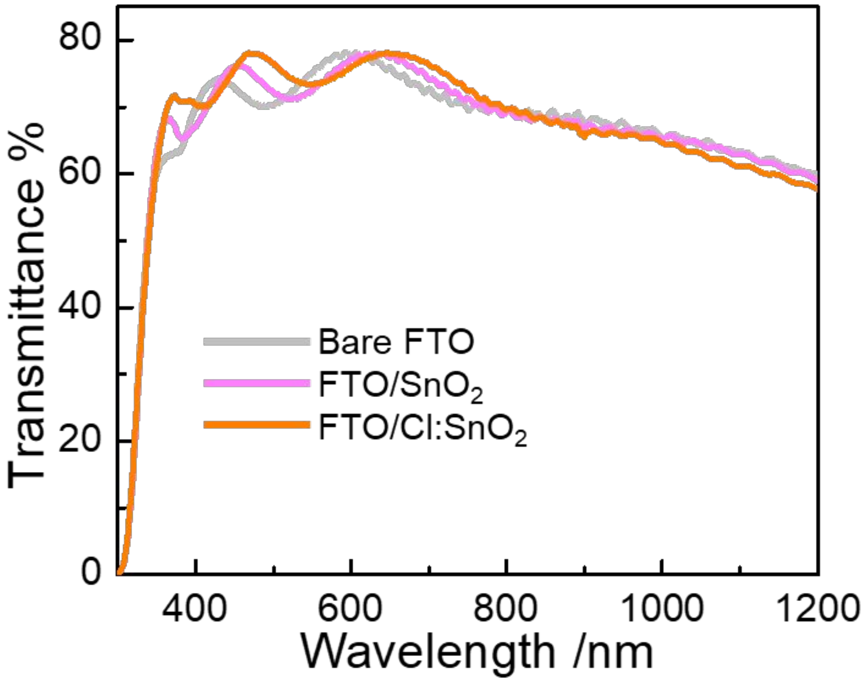

Figure S4. Transmittance spectra of the FTO glass and surface-treated FTO electrodes showing improvement in the optical transparency upon nanoparticle deposition.

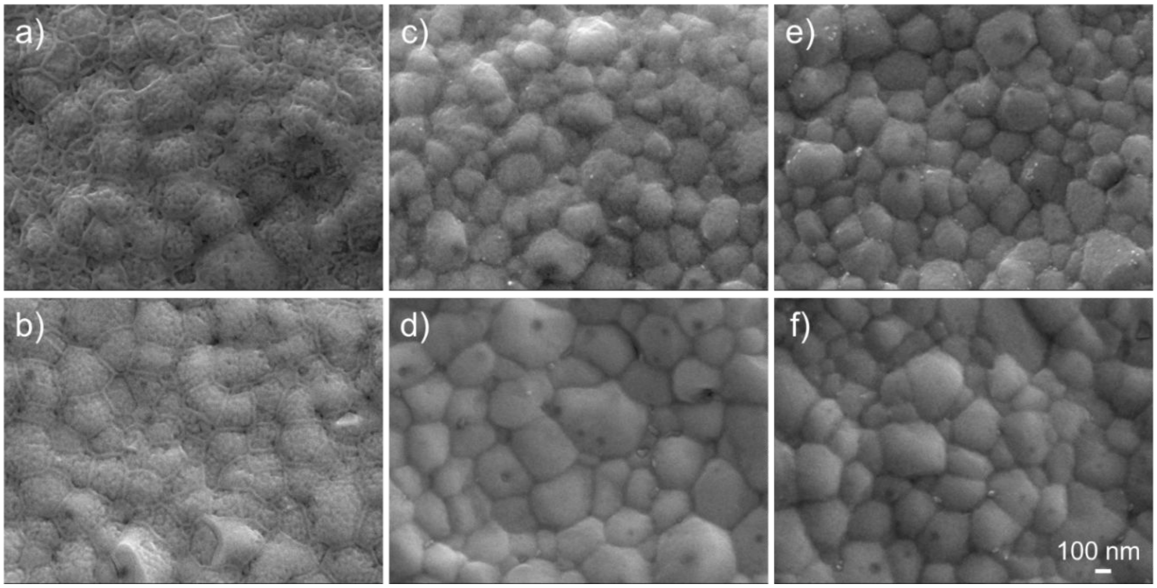

Figure S5. Surface morphologies of the perovskite samples prepared atop a) FTO (bare), b) FTO/p-Benz, c) FTO/SnO_2, d) FTO/SnO_2/p-Benz, e) FTO/Cl:SnO_2, and f) FTO/Cl:SnO_2/p-Benz electrodes.

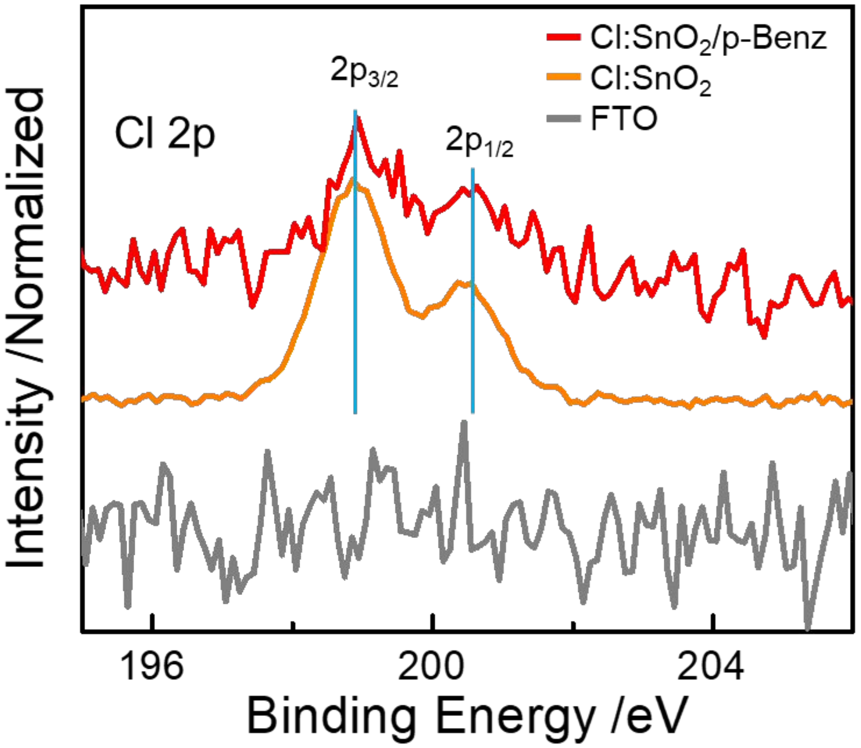

Figure S6. High resolution XPS of Cl 2p acquired from FTO, FTO/Cl:SnO_2 and FTO/Cl:SnO_2/p-Benz electrodes. The signal from Cl:SnO_2/p-Benz looks relatively weak in comparison to that from Cl:SnO_2 due to the presence of p-Benz on the surface.

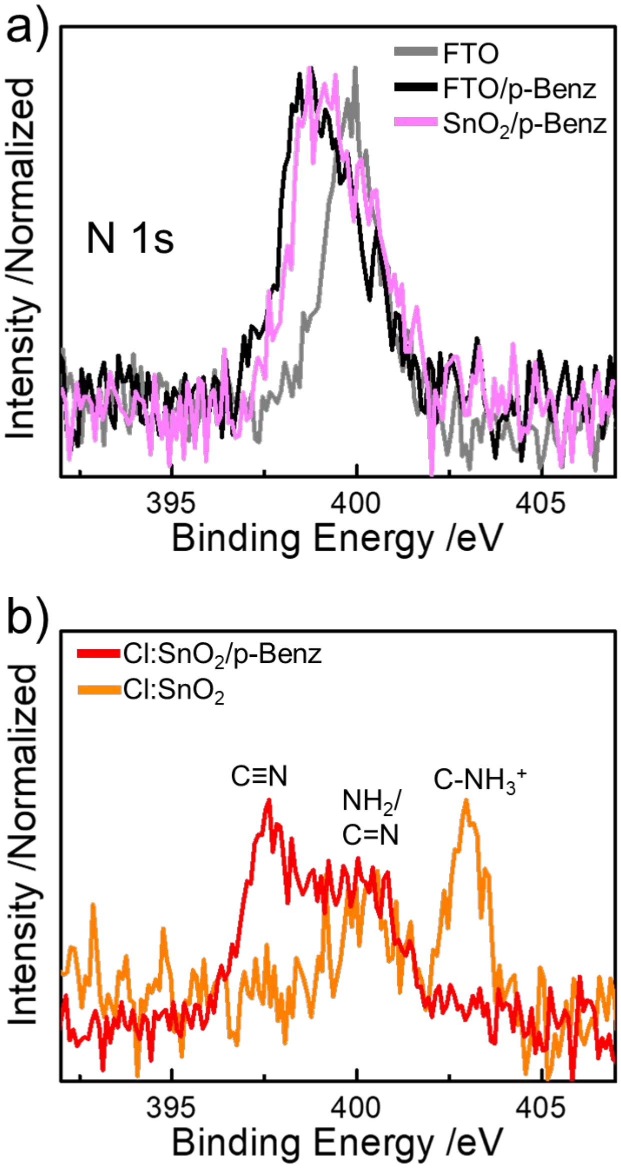

Figure S7. High resolution XPS of N 1s from a) FTO, FTO/p-Benz and FTO/SnO_2/p-Benz, and b) FTO/Cl:SnO_2 and FTO/Cl:SnO_2/p-Benz, respectively.

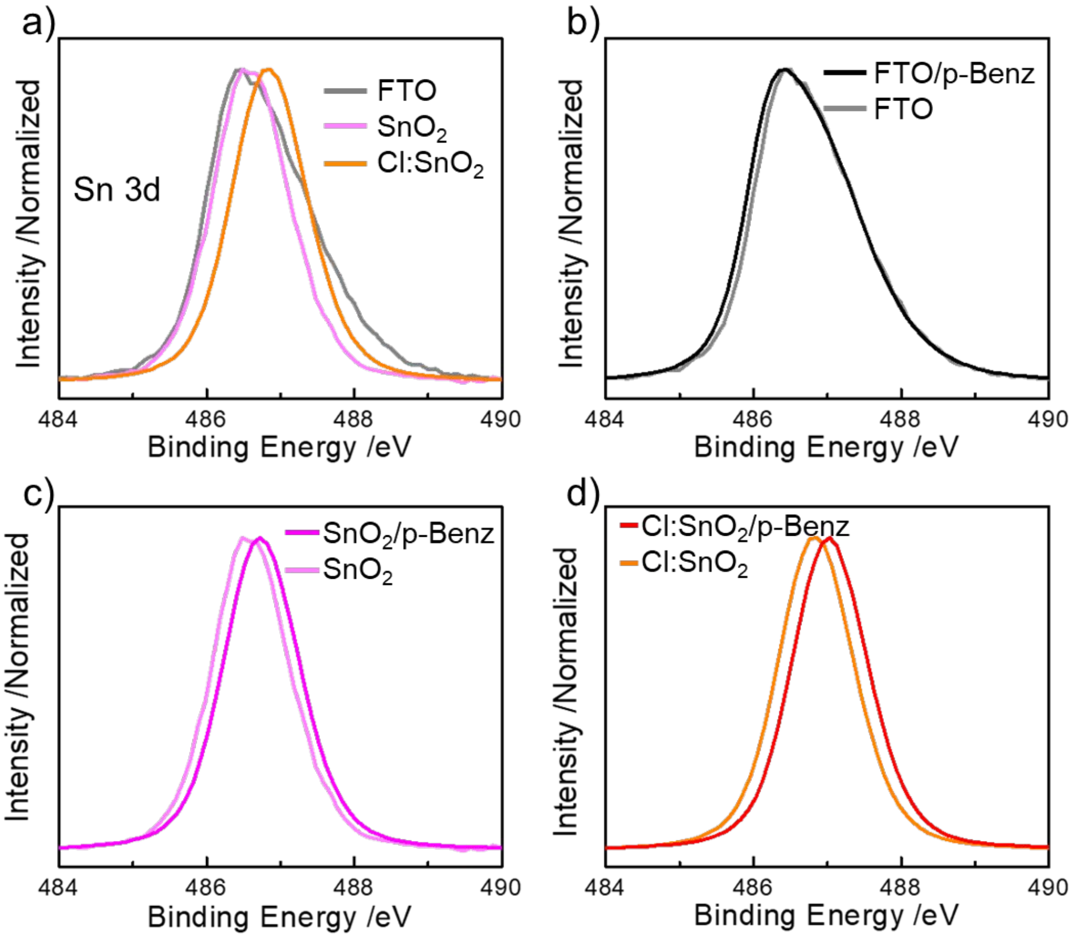

Figure S8. High resolution XPS of Sn 3d species acquired from a) FTO, FTO/SnO_2 and FTO/Cl:SnO_2, and the individual spectra showing changes between b) FTO and FTO/p-Benz, c) FTO/SnO_2 and FTO/SnO_2/p-Benz, and d) FTO/Cl:SnO_2 and FTO/Cl:SnO_2/p-Benz, respectively.

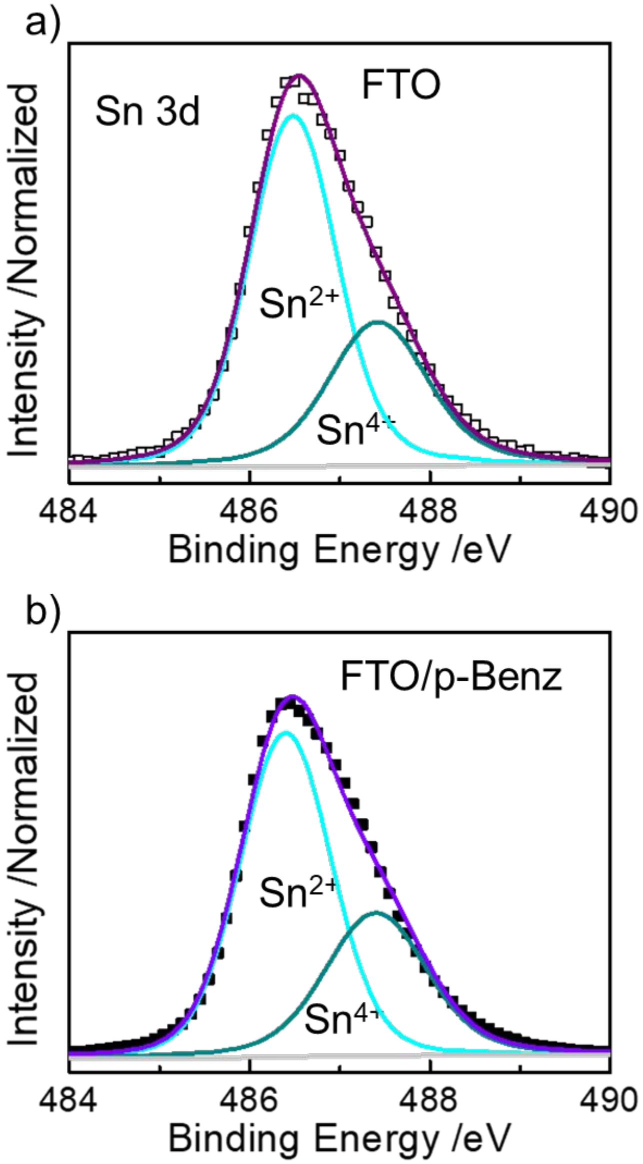

Figure S9. Sn 3d XPS from a) FTO and b) FTO/p-Benz deconvoluted to show the relative signals from Sn^{2+} and Sn^{4+}. The area ratio between Sn^{2+} and Sn^{4+} was estimated to be 65.9/34.1 and 60.7/39.3 for FTO and FTO/p-Benz, respectively.

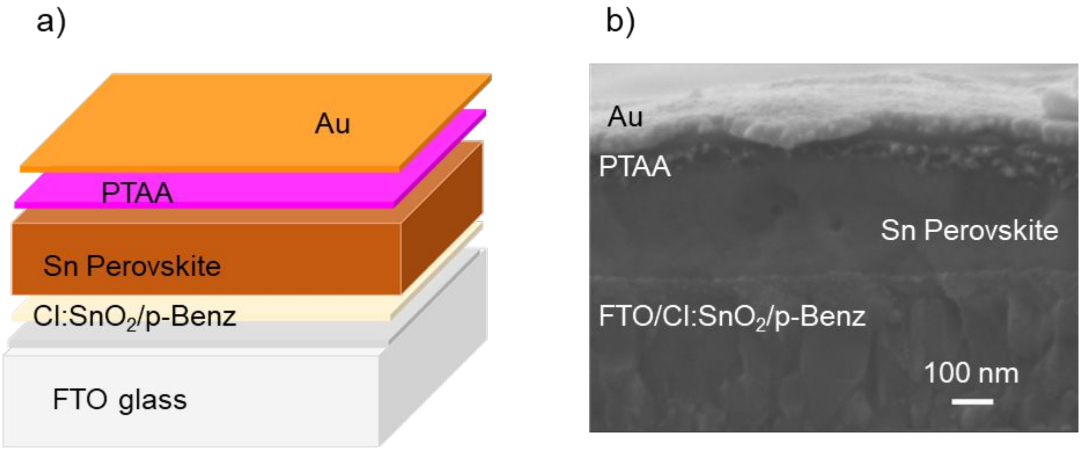

Figure S10. a) Graphical device architecture in the n-i-p configuration, and b) the cross-sectional view of the device stack using Cl:SnO_2/p-Benz.

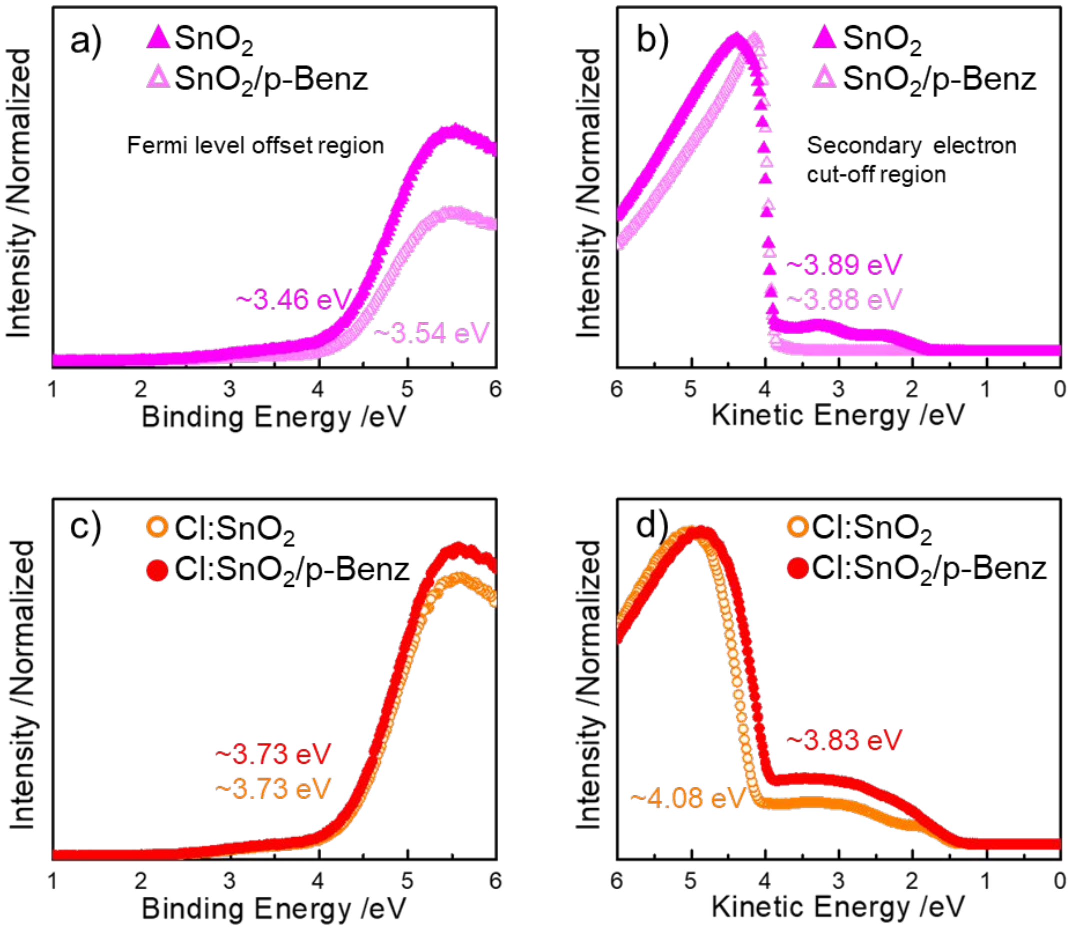

Figure S11. UPS spectra showing (a,c) the fermi level offset and (b,d) the secondary electron cut-off regions from the prepared transparent electrodes. The cut-off corresponds to the work function of the electrode (fermi level position, E_f) while the fermi level offset represents the energy difference between valence band maximum (VBM) and E_f. The source energy of photoemission is 21.2 eV (He I discharge lamp), and hence the kinetic energy shown in the figure is estimated accordingly.

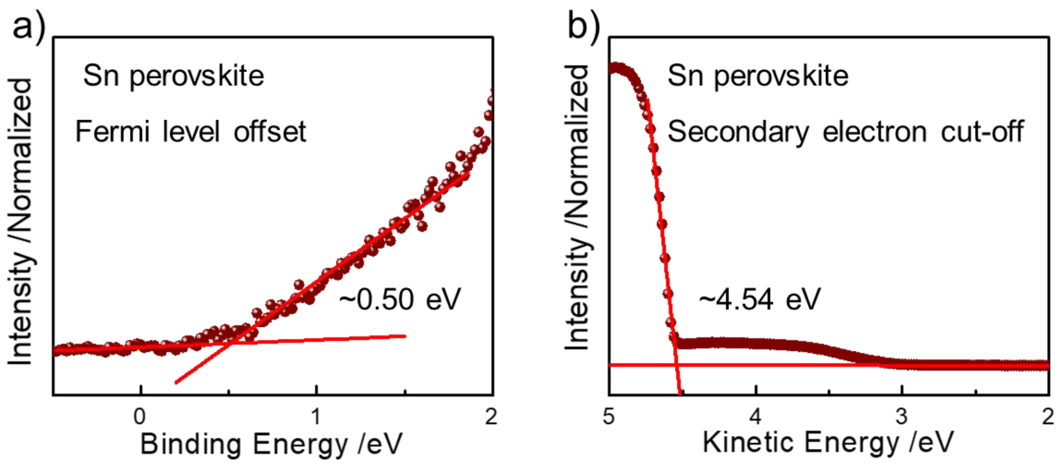

Figure S12. UPS spectra showing (a) the fermi level offset and (b) the secondary electron cut-off regions from the Sn perovskite layer. The intercepts of the linear fits corresponding to the onset of the spectra were used to extrapolate the offset and cut-off values as indicated. The VBM and E_f are estimated to be -5.04 eV and -4.54 eV, respectively, implying that the perovskite is p-type.

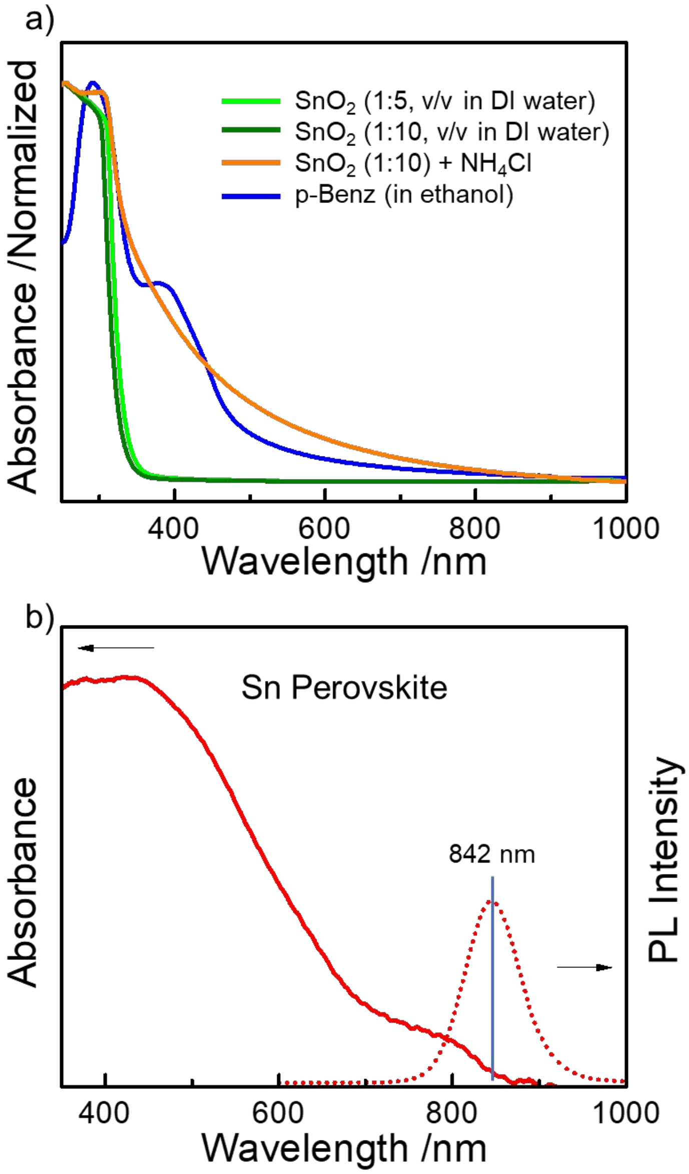

Figure S13. a) Normalized UV-visible absorbance spectra of SnO_2 colloidal dispersion diluted to different concentrations (1:5 and 1:10 v/v with DI water), SnO_2 colloidal dispersion (dilution ~1:10) with NH_4Cl (2.5 mg) as additive, and p-Benz dispersed in purified ethanol; b) UV-visible absorbance and the steady-state PL spectra recorded from the Sn perovskite sample formed on FTO glass. The band gap of SnO_2 was estimated to be ~3.79 eV, while the band gap of Sn perovskite was estimated to be ~1.47 eV (corresponding to the PL peak maximum).

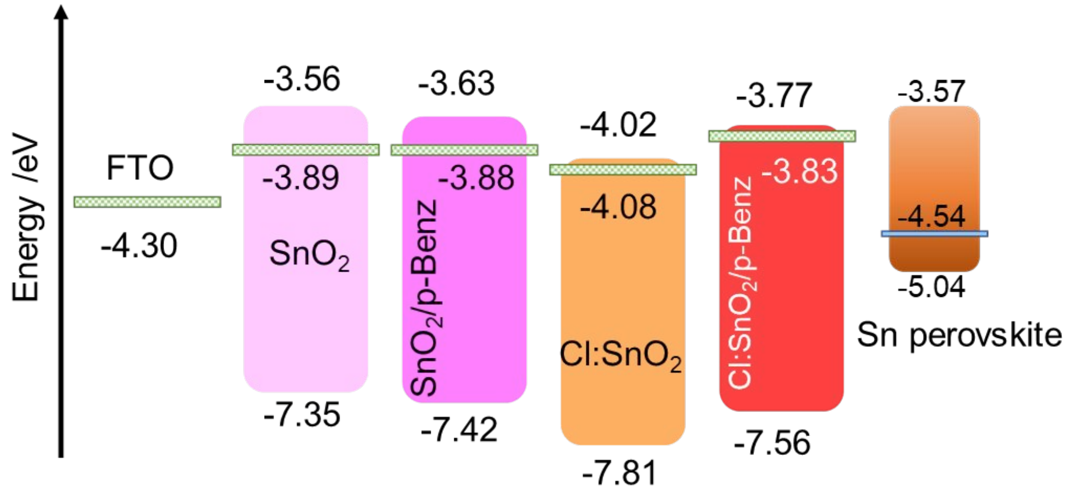

Figure S14. Energy level alignment between Sn perovskite and the transparent electrodes, based on the UPS results. The energy level difference between the conduction band minimum and the fermi level was estimated to be -0.33, -0.25, -0.06, and -0.06 eV for SnO_2, SnO_2/p-Benz, Cl:SnO_2, and Cl:SnO_2/p-Benz electrodes, respectively.

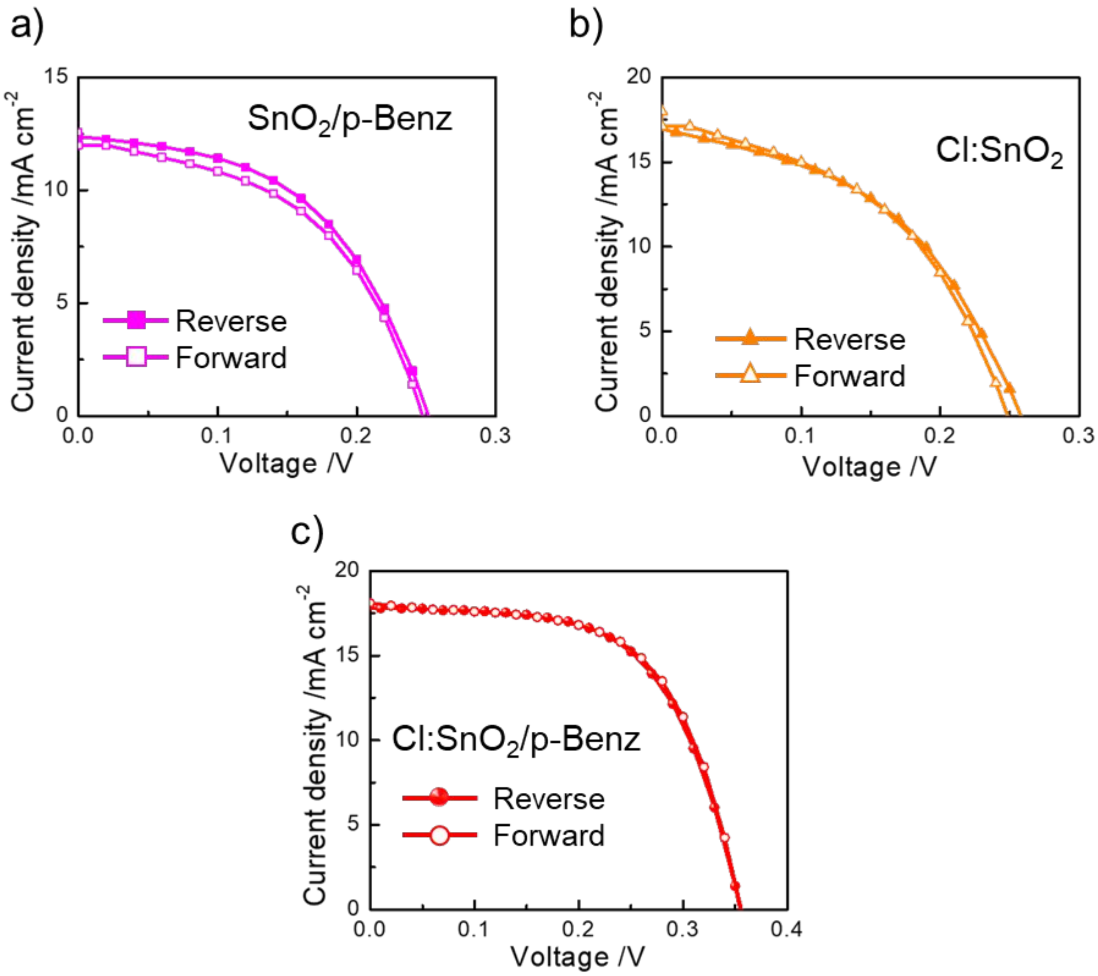

Figure S15. Reverse and forward *J-V* scans recorded from the devices based on a) SnO_2/p-Benz, b) Cl:SnO_2, and c) Cl:SnO_2/p-Benz electrodes showing hysteresis under simulated AM 1.5G illumination. The combination of both Cl and N-decorated p-Benz at the buried interface endow Cl:SnO_2/p-Benz device with well-managed ion migration evidenced from suppressed hysteresis.

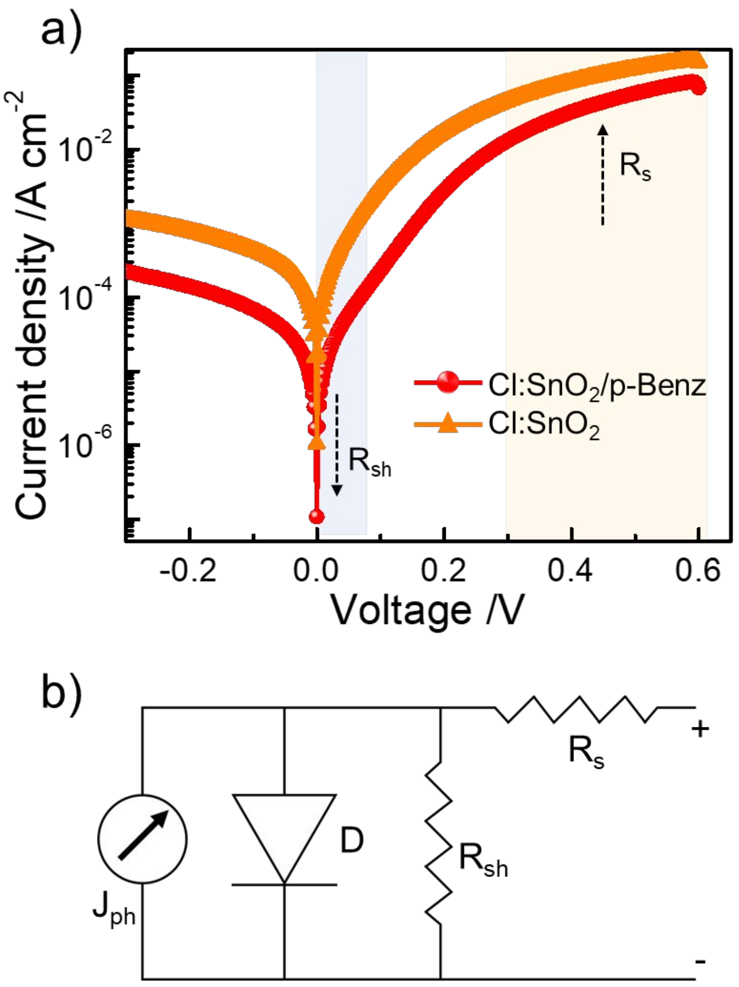

Figure S16. a) *J-V* characteristic curves (semi-log) recorded under darkness from Cl:SnO_2 and Cl:SnO_2/p-Benz devices; b) Solar cell equivalent circuit with parasitic resistances (series resistance, R_s; and shunt resistance, R_{sh}) based on single diode model. From the inverse slope of the dark *J-V* curves near the R_{sh} region, we estimated the R_{sh} to be ~1173.71 Ω cm^2 and ~119.05 Ω cm^2 for Cl:SnO_2/p-Benz and Cl:SnO_2 devices, respectively. The inverse slope near the R_s region yielded an R_s of ~2.92 Ω cm^2 and ~3.61 Ω cm^2 for Cl:SnO_2/p-Benz and Cl:SnO_2 devices, respectively.

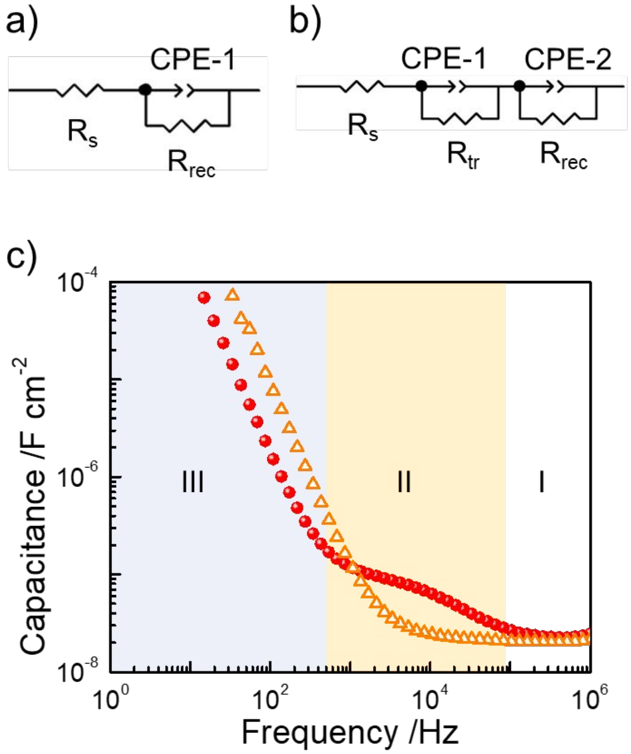

Figure S17. Equivalent circuits used to fit the Nyquist plots shown in Figures 4a,b corresponding to the device fabricated using a) FTO/Cl:SnO_2, and b) FTO/Cl:SnO_2/p-Benz electrodes; c) Capacitance vs Frequency plot derived from the Nyquist plots showing the capacitance plateau region attesting the presence and influence of p-Benz functionalization in Cl:SnO_2/p-Benz based device. Regions I and III are influenced by Rs and electrode polarization, respectively, while the capacitance plateau region (region II) occurs due to dielectric relaxation.[4]

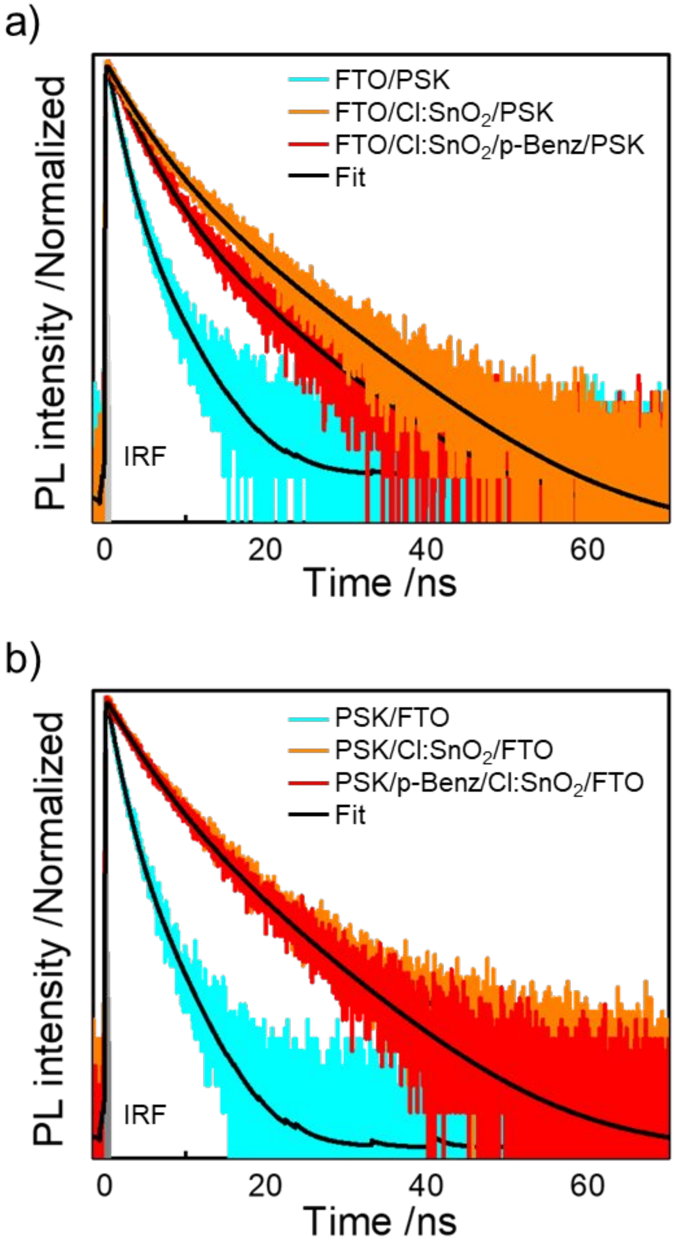

Figure S18. PL decay transients obtained from Sn perovskite samples in the absence of PTAA HTL excited from a) FTO-side, and b) Sample-side, respectively. The transients were fitted using bi-exponential functions and the fitting coefficients are shown in Tables S2 and S3.

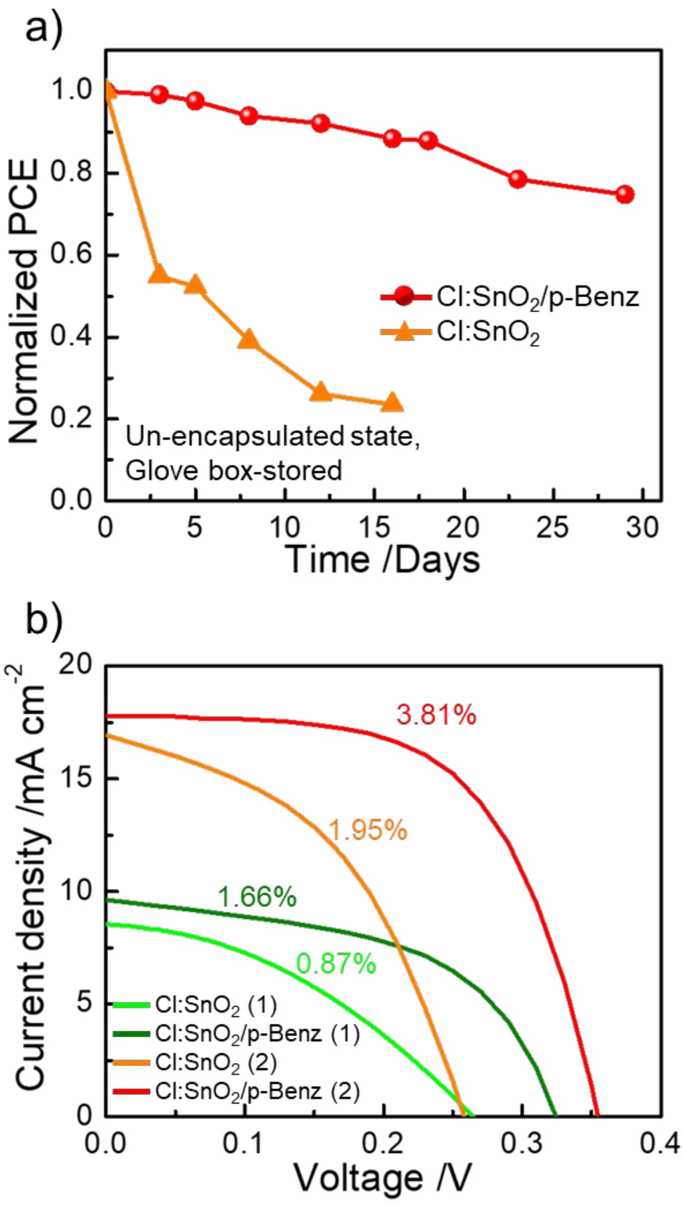

Figure S19. a) Long-term stability of Cl:SnO_2 and Cl:SnO_2/p-Benz devices upon stored in a N_2-filled glove box; b) *J-V* characteristic curves under simulated AM 1.5G illumination comparing the device performance when fabricated by applying traditional anti-solvent-mediated one-step method, and two-step sequential deposition method as marked. For one-step method, we followed an earlier report with slight modifications to get the identical perovskite composition reported in the present work.[5]

Table S1. Fitting results for the Nyquist plots shown in Figure 4a extracted from the equivalent circuits shown in Figure S17a,b. The recombination time constant was estimated to be 107.76 μs and 570.41 μs for Cl:SnO_2 and Cl:SnO_2/p-Benz device, respectively.

Device	R_s /Ω	R_{tr} /Ω	CPE-1		R_{rec} /kΩ	CPE-2	
			T	P		T	P
Cl:SnO_2	71.96	-	-		20.03	5.38E-9	0.96
Cl:SnO_2/p-Benz	43.40	832.11	1.66E-8	0.93	23.53	2.44E-8	0.94

R_s: Series resistance, R_{tr}: Charge transport resistance, R_{rec}: Recombination resistance, CPE: Constant phase element

Table S2. Fitting coefficients for the PL decay transients shown in Figure S18a.

Sample	$\alpha_1\tau_1$	$\alpha_2\tau_2$	τ_{avg}
FTO/PSK	(0.19) 4.00	(0.81) 1.36	2.44
FTO/Cl:SnO_2/PSK	(0.69) 3.81	(0.31) 10.54	7.54
FTO/Cl:SnO_2/p-Benz/PSK	(0.80) 3.23	(0.20) 9.70	6.00

Table S3. Fitting coefficients for the PL decay transients shown in Figure S18b.

Sample	$\alpha_1\tau_1$	$\alpha_2\tau_2$	τ_{avg}
PSK/FTO	(0.21) 3.70	(0.79) 1.32	2.34
PSK/Cl:SnO_2/FTO	(0.81) 4.61	(0.19) 12.64	7.75
PSK/p-Benz/Cl:SnO_2/FTO	(0.67) 4.05	(0.33) 9.78	7.16

Table S4. Fitting coefficients for the PL decay transients shown in Figure 4c.

Sample	$\alpha_1\tau_1$	$\alpha_2\tau_2$	$\alpha_3\tau_3$	τ_{avg}
FTO/PSK/PTAA	(0.70) 2.33	(0.30) 5.72	-	4.07
FTO/Cl:SnO_2/PSK/PTAA	(0.68) 3.25	(0.32) 9.77	-	7.07
FTO/Cl:SnO_2/p-Benz/PSK/PTAA	(0.68) 3.71	(0.32) 8.73	-	6.35

Table S5. Fitting coefficients for the PL decay transients shown in Figure 4d.

Sample	$\alpha_1\tau_1$	$\alpha_2\tau_2$	$\alpha_3\tau_3$	τ_{avg}
PTAA/PSK/FTO	(0.63) 2.58	(0.37) 5.66	-	4.31
PTAA/PSK/Cl:SnO_2/FTO	(0.73) 4.68	(0.27) 10.30	-	7.20
PTAA/PSK/p-Benz/Cl:SnO_2/FTO	(0.50) 0.66	(0.40) 4.34	(0.10) 9.13	5.40

REFERENCES

(1) Liu, Z.; Deng, K.; Hu, J.; Li, L. Coagulated SnO_2 Colloids for High-Performance Planar Perovskite Solar Cells with Negligible Hysteresis and Improved Stability. *Angew. Chemie - Int. Ed.* **2019**, *58* (33), 11497–11504. https://doi.org/10.1002/anie.201904945.

(2) Shahbazi, S.; Li, M. Y.; Fathi, A.; Diau, E. W. G. Realizing a Cosolvent System for Stable Tin-Based Perovskite Solar Cells Using a Two-Step Deposition Approach. *ACS Energy Lett.* **2020**, *5* (8), 2508–2511. https://doi.org/10.1021/acsenergylett.0c01190.

(3) Narra, S.; Jokar, E.; Pearce, O.; Lin, C.; Fathi, A.; Diau, E. W. Femtosecond Transient Absorption Spectra and Dynamics of Carrier Relaxation of Tin Perovskites in the Absence and Presence of Additives. *J. Phys. Chem. Lett.* **2020**, *11*, 5699–5704. https://doi.org/10.1021/acs.jpclett.0c01589.

(4) Almora, O.; Aranda, C.; Mas-Marzá, E.; Garcia-Belmonte, G. On Mott-Schottky Analysis Interpretation of Capacitance Measurements in Organometal Perovskite Solar Cells. *Appl. Phys. Lett.* **2016**, *109*, 173903. https://doi.org/10.1063/1.4966127.

(5) Jokar, E.; Chien, C. H.; Fathi, A.; Rameez, M.; Chang, Y. H.; Diau, E. W. G. Slow Surface Passivation and Crystal Relaxation with Additives to Improve Device Performance and Durability for Tin-Based Perovskite Solar Cells. *Energy Environ. Sci.* **2018**, *11* (9), 2353–2362. https://doi.org/10.1039/c8ee00956b.
